# Multimodal Ageing Biomarkers and Plasma Proteomic Signatures Associated With All‐Cause Mortality

**DOI:** 10.1111/acel.70747

**Published:** 2026-10-02

**Authors:** Maira Pyrgioti, Ines Mesa Eguiagaray, Paul Redmond, Janie Corley, Mark E. Bastin, Maria Valdés Hernández, Tom C. Russ, Joanna M. Wardlaw, Eilis Hannon, Ian J. Deary, Keenan A. Walker, Elliot M. Tucker‐Drob, Simon R. Cox, Riccardo E. Marioni, Sarah E. Harris

**Affiliations:** ^1^ Lothian Birth Cohorts, Edinburgh Futures Institute The University of Edinburgh Edinburgh UK; ^2^ Lothian Birth Cohorts, Department of Psychology The University of Edinburgh Edinburgh UK; ^3^ Institute of Genetics and Cancer The University of Edinburgh Edinburgh UK; ^4^ Institute for Neuroscience and Cardiovascular Research The University of Edinburgh Edinburgh UK; ^5^ Scottish Imaging Network A Platform for Scientific Excellence (SINAPSE) Collaboration Edinburgh UK; ^6^ Row Fogo Centre for Research Into Small Vessel Diseases Edinburgh UK; ^7^ Alzheimer Scotland Dementia Research Centre The University of Edinburgh Edinburgh UK; ^8^ Neuroprogressive and Dementia Network NHS Research Scotland Edinburgh UK; ^9^ BHF‐UK DRI Centre for Vascular Dementia Research UK Dementia Research Institute Edinburgh UK; ^10^ Department of Clinical and Biomedical Sciences University of Exeter Medical School, University of Exeter, RILD Building, Royal Devon & Exeter Hospital Exeter Devon UK; ^11^ Laboratory of Behavioral Neuroscience National Institute on Aging Baltimore Maryland USA; ^12^ Department of Psychology The University of Texas Austin Texas USA

## Abstract

Ageing biomarkers quantify molecular, physiological or functional features that vary between individuals as they age and may improve prediction of health outcomes beyond chronological age. Recently, plasma proteomics have been used to estimate the biological ages of 11 human organs. Although accelerated organ ageing is associated with higher mortality risk, systematic benchmarking against established ageing biomarkers is lacking. Here, we pursued two complementary aims. First, we benchmarked proteomic organ clocks against multimodal ageing biomarkers for associations with 16‐year all‐cause mortality in the Lothian Birth Cohort 1936 (LBC1936) using Cox regression (861 participants; 444 deaths). Biomarkers included the epigenetic clock GrimAge2, telomere length, physical function (grip strength, walk time and respiratory function), neuroimaging and cognitive measures. Among proteomic clocks, accelerated liver (HR_perSD_ [95% CI] = 1.43 [1.30–1.58]), immune (1.42 [1.29–1.57]) and heart (1.38 [1.25–1.53]) ageing were most strongly associated with increased mortality risk. However, older epigenetic age, smaller total brain and grey matter volumes, reduced respiratory function and poorer cognition showed stronger associations with mortality (HR_perSD_ = 1.44–1.62). Second, survival analyses of 9703 plasma protein targets identified 368 candidates associated with mortality, with high‐risk proteins enriched for immune functions, and low‐risk proteins involved in genomic stability and cellular maintenance. GDF15 (HR_perSD_ [95% CI] = 1.56 [1.42–1.72]), WFDC2 (1.47 [1.33–1.62]) and TIMP1 (1.45 [1.29–1.62]) were the leading proteomic candidates associated with mortality. This study provides a systematic, cross‐modal benchmarking of proteomic organ clocks against established ageing biomarkers and identifies proteins and biological functions associated with mortality risk.

## Graphical abstract

We benchmarked 23 ageing biomarkers and 9703 protein targets for associations with mortality risk. Epigenetic, neuroimaging and functional biomarkers showed the strongest associations, outperforming proteomic organ clocks. Among 368 mortality‐associated proteins, high‐risk proteins were enriched in immune functions, whereas low‐risk proteins were enriched in genomic integrity and cellular maintenance processes.

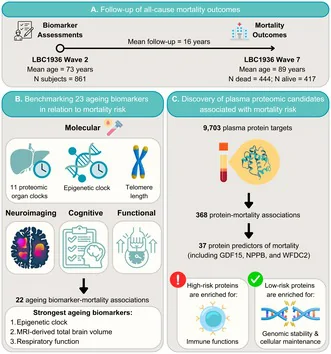

## 1 Introduction

Ageing is characterised by the progressive accumulation of molecular and cellular damage, resulting in declining physical and cognitive capacity, increased disease risk and ultimately, death (Jylhävä et al. 2017; López‐Otín et al. 2023; Mathur et al. 2024). Chronological age measures the time elapsed since birth; however, individuals of the same chronological age can differ substantially in functional capacity and health status because of variation in genetics, lifestyle, environmental exposures and stochastic processes (Salih et al. 2023). In contrast, biological age captures an individual's current physiological state and may therefore improve prediction of future health outcomes beyond chronological age (Jylhävä et al. 2017). The development of reliable ageing biomarkers is becoming increasingly important as the global population ages, with the World Health Organization projecting that the number of people aged 60 years and older will double to 2.1 billion by 2050 (World Health Organization (WHO) 2025). However, there is currently no consensus on how best to measure biological age, and different approaches often yield different results.

Ageing biomarkers are measurable biological parameters that correlate with chronological age and may provide additional prognostic information for health risk assessment beyond chronological age (Jylhävä et al. 2017). Over time, multiple biomarker modalities have emerged, including molecular, cellular, functional and imaging measures (Furrer and Handschin 2025; Tao et al. 2024). Telomere shortening is a well‐established hallmark of cellular ageing, reflecting cumulative replicative history and exposure to cellular stress (Levy et al. 1992). Nevertheless, it correlates only weakly with chronological age and is a noisy measure that shows inconsistent associations with lifespan across studies, with several reporting non‐significant associations (Wang et al. 2018; Bischoff et al. 2006; Martin‐Ruiz et al. 2005; Ye et al. 2023). Physical function biomarkers (such as grip strength and gait speed) are simple, inexpensive and non‐invasive, and are strong predictors of disability, frailty, diseases and mortality (De Souza et al. 2025; Gale et al. 2007; Studenski 2011). However, they typically capture ageing at relatively late stages, after substantial physiological deterioration has already occurred and provide no insight into underlying molecular mechanisms. Neuroimaging biomarkers can detect early neurodegenerative changes prior to clinical symptom onset and are strong predictors of cognitive decline, neurological diseases and mortality (Fletcher et al. 2018; Ghaznawi et al. 2021; He et al. 2025; Rensma et al. 2018; Van Elderen et al. 2016). While informative, they are expensive and resource‐intensive to generate, and, like physical function biomarkers, do not directly capture molecular processes. This highlights the need for biomarkers that are both mechanistically insightful and broadly predictive of survival.

Advances in high‐throughput omics technologies have brought a new generation of ageing biomarkers to the fore. Biological age estimates, or clocks, are derived using mathematical models trained to predict chronological age from omics data. The difference between predicted and expected biological age, referred to as the *age gap* or *age acceleration*, provides a relative measure of ageing, indicating whether an individual is ageing faster or slower than others of the same chronological age (Salih et al. 2023). Epigenetic clocks, which capture age‐related DNA methylation patterns influenced by genetic variation, environmental and lifestyle exposures, have shown robust associations with mortality risk; however, they largely provide a single, systemic measure of ageing (Hannum et al. 2013; Horvath and Raj 2018; Lu et al. 2022; Mavrommatis et al. 2025). Recent advances in aptamer‐based proteomics enable the quantification of over 10,000 plasma proteins spanning diverse tissues and biological pathways, providing a comprehensive and biologically informative readout of ageing biology (Kirsher et al. 2025). Oh et al. (2023) developed organ‐specific proteomic clocks by mapping these plasma proteins to their tissues of origin and training age predictors for 11 major organs, including the brain, heart, immune system and liver. This approach revealed substantial heterogeneity in ageing both between individuals and across organs within the same individual. Moreover, accelerated organ ageing was associated with a 17%–53% higher risk of mortality per standard deviation. However, the comparative performance of proteomic organ clocks for mortality risk assessment relative to other ageing biomarkers within the same population remains unknown. This is critical to determine whether organ clocks add value beyond existing biomarkers and to prioritise the most informative biomarkers for risk stratification.

Here, we pursue two complementary aims. First, we benchmark the 11 proteomic organ clocks against molecular, physical function, cognitive and neuroimaging ageing biomarkers for their associations with 16‐year all‐cause mortality risk in the Lothian Birth Cohort 1936 (LBC1936) (*N* = 861; 444 deaths) using Cox regression. This approach can elucidate the multi‐systemic contributions to mortality. Second, we conduct survival analyses of 9703 plasma protein targets to identify new proteomic candidates associated with longevity, and perform enrichment analyses to characterise the underlying biological processes. We further apply elastic net‐penalised Cox regression to derive a concise set of proteins associated with mortality. This integrated, multimodal benchmarking approach reveals the most informative ageing biomarkers, proteins and biological pathways underlying mortality risk.

## 2 Methods

### 2.1 Study Population

The LBC1936 is a longitudinal study of ageing trajectories in individuals born in 1936 who participated in the Scottish Mental Survey at age 11 and were residing in Scotland at the time of recruitment. Participants underwent repeated, comprehensive assessments of cognitive function, brain imaging, physical health and fluid biomarkers approximately every 3 years, between the ages of ~70 and ~89 years. SomaScan 11K (v5.0) plasma proteomics and brain imaging were introduced at wave 2 (mean age = 73 years). Mortality status was ascertained via linkage to the National Health Service Central Register (NHSCR), managed by the National Records of Scotland (NRS), which provides regular updates. The mortality outcomes used in the present study were last updated on 9 September 2025. Detailed cohort characterisation and methodologies have been described previously (Deary et al. 2012; Taylor et al. 2018). All baseline biomarker measurements for the present study were obtained from wave 2 and are summarised in Table 1. Many of these non‐organ‐age biomarkers have previously been associated with mortality in this cohort, albeit with shorter follow‐up periods and lower mortality incidence (Cole et al. 2018; Corley et al. 2019; Harris et al. 2016; Hillary et al. 2021; McLachlan et al. 2020).

**TABLE 1:** Baseline characteristics of LBC1936 wave 2.

Variables	Mean (SD)	*N* Subjects
Demographic factors		
Chronological age (years)	72.5 (0.7)	861
Male/female	N/A	445/416
Mortality ascertainment		
Time‐to‐death (years)	10.0 (4.1)	444
Time‐to‐censor (years)	16.2 (0.7)	417
Lifestyle risk factors		
Smoking pack years	15.4 (24.7)	850
Alcohol units per week	11.7 (14.4)	769
BMI (kg/m^2^)	27.9 (4.5)	861
Physical function measures		
Six meter walk time (s)	4.4 (1.3)	855
Grip strength (kg)	28.5 (9.4)	818
FEV (L)	2.3 (0.7)	851
FVC (L)	3.1 (0.9)	851
FER (%)	77.4 (11.7)	851
Brain MRI volume measures		
TBV/ICV	0.7 (0.02)	656
GMV/ICV	0.3 (0.02)	656
WMHV/ICV	0.008 (0.009)	666
Molecular measures		
Leukocyte telomere length (bp)	3969.7 (738.1)	839
Disease prevalence		
Hypertension	NA	425
Arthritis	NA	396
High cholesterol	NA	356
Cardiovascular disease	NA	250
Neoplasm	NA	130
Diabetes	NA	95
Stroke	NA	55
Parkinson's disease	NA	5
Dementia	NA	2

Abbreviations: BMI, body mass index; FER, forced expiratory ratio; FEV, forced expiratory volume in 1 s; FVC, forced vital capacity; GMV/IVC, grey matter volume to intracranial volume ratio; MRI, magnetic resonance imaging; TBV/ICV, total brain volume to intracranial volume ratio; WMHV/ICV, white matter hyperintensity volume to intracranial volume ratio.

### 2.2 Plasma Proteomics

Plasma was extracted from blood collected in citrate tubes at waves 2 (age ~73 years), 3 (age ~76 years), 5 (age ~82 years) and 6 (age ~86 years). Protein quantification was performed using the SomaScan 11K (v5.0) platform, a high‐throughput proteomic assay that employs slow off‐rate modified DNA aptamers (SOMAmers) and a DNA microarray readout to measure 11,083 protein targets in relative fluorescence units (RFU) from 55 μL of plasma (Candia et al. 2024; Kirsher et al. 2025). Standard normalisation procedures, including hybridisation control normalisation, median signal normalisation on calibrators, plate‐scale normalisation, interplate calibration and median signal normalisation on all sample types, were performed by SomaLogic as previously described (Candia 2025). Additional quality control (QC) procedures were applied to exclude non‐human and non‐protein aptamers, aptamers with a coefficient of variation (CV) greater than 50%, low‐variance aptamers, aptamers with a median RFU above 80,000, and those with signal‐to‐noise ratios below 1. Principal component analysis revealed differences between waves, particularly in wave 2. Consequently, 963 aptamers exhibiting low correlation (*r* < 0.3) and high absolute mean difference (> 0.5 units on the log2 scale) between waves were also removed. Cross‐referencing these aptamers with SomaLogic pre‐analytical variation (PAV) tables derived from the SomaScan 7k assay indicated that over 75% of these aptamers displayed moderate‐to‐high PAV effects. Table S1 summarises the QC status of all 11,083 aptamers, indicating whether they passed QC, were non‐human, were likely affected by pre‐analytical variation, had a high CV or RFU, or had a signal‐to‐noise ratio below 1. Following QC, 9703 aptamers, corresponding to 8699 unique UniProt IDs, were retained for LBC1936 wave 2 (*N* = 789). Protein expression values were log10‐transformed and standardised (mean = 0, SD = 1) prior to downstream analyses.

### 2.3 Proteomic Organ Clocks

Proteomic organ ages were derived in LBC1936 wave 2 using the organ‐specific age predictors previously established by Oh et al. (2023). The organs comprised adipose tissue, artery, brain, heart, immune system, intestine, kidney, liver, lung, muscle and pancreas. Organ‐enriched transcripts were defined as those exhibiting at least four‐fold higher expression in a given organ relative to all others, based on bulk tissue RNA‐sequencing data from the Genotype‐Tissue Expression (GTEx) Project. Tissues corresponding to the same organ were aggregated such that a gene's expression level for each organ was defined by the maximum expression observed among its constituent subtissues (e.g., all GTEx brain regions were treated as subtissues of the brain organ). The immune organ was characterised using gene expression data from blood and spleen. These organ‐enriched transcripts were subsequently mapped to 4979 plasma proteins measured by the SomaScan assay, and 893 organ‐enriched proteins were identified. After additional QC to remove proteins with a high coefficient of variation or a low correlation between SomaScan assay versions v.4 (5k panel) and v.4.1 (7k panel), 4778 proteins were retained, corresponding to 856 organ‐enriched proteins. Organ‐specific age predictors were trained in 1398 cognitively normal participants from the Knight Alzheimer Disease Research Center (Knight‐ADRC) cohort. For each organ, bootstrap‐aggregated LASSO regression models (500 bootstrap iterations per organ) were fitted with standardised (mean = 0, SD = 1), log10‐transformed organ‐enriched protein levels as predictors, chronological age as the outcome, and sex included as a covariate. For LBC1936 wave 2, log10‐transformed SomaScan 11K protein measures were standardised (mean = 0, SD = 1) and aligned to the protein coefficients published by Oh et al. (2023). For each participant, predicted organ age was calculated by applying the 500 bootstrap sets of protein and sex coefficients and averaging the resulting predicted ages (bagging) to obtain a single age estimate per organ. Organ age gaps were defined as standardised (mean = 0, SD = 1) residuals from linear regression models of predicted organ age on chronological age, fitted separately for each organ. Positive age gaps indicate relatively accelerated organ ageing and negative values indicate relatively slower organ ageing compared with peers of the same chronological age.

### 2.4 GrimAge2 Acceleration

DNA methylation (DNAm) profiling was performed on whole‐blood LBC1936 wave 2 samples using the Illumina HumanMethylation450K BeadChip. Detailed protocols for quantification and QC of DNAm data in the LBC1936 have been reported previously (Zhang et al. 2018). DNAm GrimAge2 was estimated for LBC1936 samples using Horvath's online calculator (https://dnamage.clockfoundation.org/). GrimAge2 was developed via a two‐stage process (Lu et al. 2022). First, DNAm‐based surrogates were established for smoking pack‐years and nine plasma proteins: adrenomedullin (ADM), beta‐2 microglobulin (B2M), cystatin C (CST3), growth differentiation factor 15 (GDF15), leptin (LEP), plasminogen activator inhibitor‐1 (PAI‐1), tissue inhibitor of metalloproteinases 1 (TIMP1), log‐transformed high‐sensitivity C‐reactive protein (CRP) and log‐transformed haemoglobin A1C (HbA1c). Second, a Cox regression model with elastic net regularisation was used to predict all‐cause mortality, with time‐to‐death as the outcome and the nine DNAm protein surrogates, DNAm smoking pack‐years, chronological age and sex as predictors. The resulting uncalibrated DNAm GrimAge2 estimate was linearly transformed to units of years to serve as a physiological age estimator. GrimAge2 acceleration was calculated as the standardised residuals from a linear model regressing GrimAge2 on chronological age, with positive values indicating accelerated ageing and negative values indicating decelerated ageing.

### 2.5 Telomere Length

DNA was extracted from peripheral blood leukocytes, and telomere length was measured by quantitative real‐time polymerase chain reaction (qPCR) using an Applied Biosystems 7900HT Fast Real‐Time PCR system (Pleasanton, CA, USA) (Martin‐Ruiz et al. 2005). Four internal control DNA samples were run within each plate to calculate absolute telomere lengths and to account for inter‐plate variation. These controls consisted of cell lines with known telomere lengths of 6.9 kb, 4.03 kb, 2.0 kb and 1.32 kb. The relative telomere‐to‐glyceraldehyde 3‐phosphate dehydrogenase (T/S) ratios of the controls were used to generate a standard regression line, which was then applied to convert the relative telomere length measurements of study samples into absolute telomere lengths. This methodology has been described in detail previously (Harris et al. 2016).

### 2.6 Physical Function

Methods for measuring physical function biomarkers have been described previously (Deary et al. 2007; Okely and Deary 2020). Walk time was assessed as the time (in seconds) taken to walk 6 m along a corridor, measured with a stopwatch, with longer times indicating slower walking (Okely and Deary 2020). Grip strength was measured three times in each hand using a North Coast Hydraulic Hand Dynamometer (JAMAR) (Deary et al. 2007; Okely and Deary 2020), and the highest score from the three attempts with the dominant hand was used. Lung function was assessed using a Micro Medical Spirometer and included forced expiratory volume (FEV, L), defined as the volume of air expelled during the first second of a forceful exhalation; forced vital capacity (FVC, L), the total volume of air expelled during a forceful exhalation; and the forced expiratory ratio (FER, %), representing the percentage of air exhaled in the first second relative to the total exhaled volume (Deary et al. 2007; Okely and Deary 2020). Participants were given three attempts on the spirometer, and the highest scoring attempt was used.

### 2.7 General Cognitive Function (*g*)

LBC1936 wave 2 participants completed 13 cognitive tests spanning four cognitive domains, as described in detail elsewhere (Deary et al. 2007; Moodie et al. 2026; Ritchie et al. 2016; Tucker‐Drob et al. 2014). The domains and corresponding tests were as follows:

1. Visuospatial Skills—Block Design (Wechsler 1997a), Matrix Reasoning (Wechsler 1997a) and Spatial Span (Wechsler 1997b).
2. Processing Speed—Symbol Search (Wechsler 1997a), Digit‐Symbol Substitution (Wechsler 1997a), Inspection Time (Deary et al. 2004) and Four‐Choice Reaction Time (Deary et al. 2001).
3. Verbal Memory—Verbal Paired Associates (Wechsler 1997b), Logical Memory (Wechsler 1997b) and Digit Span Backwards (Wechsler 1997a).
4. Crystallised Ability—Verbal Fluency (Lezak et al. 2004), National Adult Reading Test (NART; Nelson and Wilson 1991) and Wechsler Test of Adult Reading (WTAR; Wechsler 2001).

All cognitive test scores were standardised (mean = 0, SD = 1) prior to analysis to ensure comparable scaling of factor loadings across measures. General cognitive function (*g*) was estimated using structural equation modelling (SEM) implemented in the lavaan R package (version 0.6.19) (Rosseel 2012) and the R code for deriving *g* has been previously described (Moodie et al. 2024). A single latent factor model was specified in which all 13 cognitive tests loaded onto a common latent factor representing *g*. Choice Reaction Time was specified with a negative factor loading so that higher latent *g* scores consistently reflected better cognitive performance across all measures. To account for shared variance among tests within the same cognitive domain that was not attributable to *g*, residual covariances were specified between tests within each cognitive domain. The SEM was fitted using full information maximum likelihood (FIML) estimation to handle missing data under the assumption of missing at random. Individual‐level *g* scores were derived as empirical Bayes estimates of the latent factor using the fitted SEM.

### 2.8 Brain MRI Measures

MRI data acquisition and processing for the LBC1936 have been detailed previously (Wardlaw et al. 2011). Briefly, participants underwent whole‐brain MRI on the same 1.5 T GE Signa Horizon HDx clinical scanner (General Electric, Milwaukee, WI, USA), equipped with a self‐shielded gradient system (maximum gradient strength 33 mT/m) and a manufacturer supplied eight‐channel phased‐array head coil. The structural imaging protocol included whole‐brain T1‐weighted, T2‐weighted, T2*‐weighted and fluid‐attenuated inversion recovery (FLAIR) sequences. White matter hyperintensities (WMH) were defined as diffuse, patchy areas in the deep or periventricular white matter, basal ganglia or brain stem, measuring > 3 mm in diameter. These regions are hyperintense with respect to normal‐appearing grey and white matter on T2‐weighted and FLAIR, but not as hyperintense as cerebrospinal fluid (CSF) on T2‐weighted images. Quantification of WMH at wave 2 was performed using a semi‐automated threshold‐based segmentation pipeline that fuses FLAIR and T2*‐weighted MRI sequences—after mapping both to the red‐green colour space—and applies minimum variance quantisation to the multispectral colour image space to improve tissue discrimination.

Intracranial volume (ICV) was extracted and measured on the T2*‐weighted image using the Object Extraction Tool in Analyze 9.0, as previously described (Zhan et al. 2015). Following ICV extraction, brain tissue segmentation into grey matter, white matter, and CSF was performed on T1‐weighted images using FAST (FMRIB's Automated Segmentation Tool) from the FMRIB Software Library (Zhang et al. 2001). To minimise misclassification of WMH, FAST‐derived tissue segmentations were corrected using WMH binary masks, ensuring that WMH were treated as a distinct tissue class. Further refinement of misclassified dura as normal‐appearing white matter was carried out aided by the colour combination of the T1‐weighted and T2‐weighted images. Regions corresponding to old infarcts were identified using a semi‐automated procedure (Hernandez et al. 2012) and excluded from all tissue masks prior to volumetric analyses.

CSF volume comprised all fluid within the cranial cavity, including the ventricles and superficial subarachnoid space. Dura and venous sinuses were separated from the non‐brain tissue within the ICV by the T1‐weighted‐T2‐weighted combination mentioned above. Total brain volume (TBV) was defined as ICV minus CSF volume, dura and venous sinuses. Grey matter volume (GMV) included cortical and deep grey matter and was derived by subtracting white matter, stroke lesions and WMH masks from the TBV mask. The proportions of TBV, GMV and WMH volume relative to ICV were calculated and used in statistical analyses to normalise volumetric measures and account for inter‐individual differences in head size (Aribisala et al. 2013). All segmentation results were checked for accuracy and manually corrected when/if required.

MRI‐derived brain age was predicted using brainageR v2.1 (R code: https://github.com/james‐cole/brainageR) (Cole et al. 2018). In brief, T1‐weighted MRI scans were segmented into grey matter (GM) and white matter (WM) and subsequently normalised in common space using non‐linear spatial registration. The normalised GM and WM images were concatenated and converted into a similarity matrix of training subjects' data, which was used to predict chronological age using Gaussian Process regression. Model performance was assessed using 10‐fold cross‐validation, comparing MRI‐derived brain age with chronological age (*r* = 0.94). Model coefficients learned during training on the brains of 2001 healthy adults were then applied to T1‐weighted MRI scans acquired from 662 LBC1936 wave 2 participants to predict brain age. The MRI‐derived brain age gap was defined as the standardised residual from a linear regression of MRI‐derived brain age on chronological age.

### 2.9 Disease Prevalence

Disease prevalence at LBC1936 wave 2 was assessed using self‐reported diagnoses. To ensure stable estimates in survival models, only diseases with more than 50 prevalent cases were included as covariates. These included hypertension, arthritis, hypercholesterolaemia, cardiovascular disease, neoplasm, diabetes and stroke. Dementia and Parkinson's disease were excluded due to their low prevalence (≤ 5 cases).

### 2.10 Statistical Analyses

All analyses were performed using R version 4.4.2 (R Core Team 2024). ICV‐normalised WMH volume, smoking pack years, and alcohol units per week were natural log‐transformed [log(x + 1)] due to right skewness and zero inflation. All continuous variables were subsequently standardised (mean = 0, SD = 1). All *p*‐values were adjusted for multiple comparisons using the Benjamini‐Hochberg false discovery rate (FDR) procedure as implemented in the stats package in R (Benjamini and Hochberg, 1995; R Core Team 2024), and results were considered statistically significant at *p*_FDR_ < 0.05.

#### 2.10.1 Correlations

Pairwise correlations among ageing biomarkers were computed using Pearson correlation coefficients with *Hmisc* (version 5.2.2) (Harrell 2025) and visualised with *corrplot* (version 0.95) (Wei and Simko 2024).

#### 2.10.2 Survival Analyses

Cox proportional hazards models were used to examine associations between 23 ageing biomarkers and 9703 plasma protein targets, all measured at wave 2, and all‐cause mortality in LBC1936 participants followed for an average of 16 years, using the survival R package (version 3.8.3) (Therneau 2024; Therneau and Grambsch 2000). Time‐to‐event was defined as the interval (in years) between biomarker assessment at wave 2 and the date of death or last follow‐up (censoring). Hazard ratios (HRs) represent the change in mortality risk per one standard deviation increase in the predictor. Biomarkers negatively associated with mortality were labelled with a negative sign in figures, and their reciprocal HRs (1/HR) were plotted to enable effect size comparisons. Proportional hazards assumptions were evaluated through Schoenfeld residuals using the cox.zph function from the survival package. For associations violating the proportional hazards assumption (Schoenfeld residuals *p* < 0.05), sensitivity analyses were performed across yearly follow‐up intervals.

For each biomarker, three separate Cox models were fitted with incremental adjustment for: (1) demographic factors (age and sex), (2) lifestyle risk factors (smoking pack years, alcohol units per week and BMI) and (3) seven chronic medical conditions (hypertension, arthritis, hypercholesterolaemia, cardiovascular disease, neoplasm, diabetes and stroke). Plasma protein models were additionally adjusted for kidney function (estimated glomerular filtration rate, eGFR) in the second model. eGFR was calculated from serum creatinine levels, chronological age and sex according to the CKD‐EPI Creatinine Equation (National Kidney Foundation 2021; Inker et al. 2021).

Additionally, a multivariable Cox proportional hazards model including 21 ageing biomarkers simultaneously was fitted to determine whether each biomarker was associated with all‐cause mortality independently of the others after accounting for shared variance between them. This model was adjusted for demographic and lifestyle risk factors. Highly collinear biomarker pairs (Pearson's *r* > 0.7, *p*_FDR_ < 0.05; FER‐FEV and GMV‐TBV) were handled by randomly excluding one biomarker from each pair, resulting in 21 biomarkers included in this saturated model. Furthermore, Nagelkerke's pseudo‐*R* ^2^ was calculated to compare the explanatory power of Cox models containing different ageing biomarker sets, providing a measure of the variation in all‐cause mortality accounted for by each model.

To address the high dimensionality and multicollinearity in proteomics, we further ran an elastic net‐penalised Cox regression on all 9703 protein targets simultaneously using the *glmnet* R package (version 4.1.8) (Friedman et al. 2010; Tay et al. 2023). The mixing parameter (α) was set to 0.5, and the penalty parameter (λ) was chosen as 0.16 based on the λ_min_ from 5‐fold cross‐validation. Alive and dead subjects were evenly distributed across folds.

#### 2.10.3 Enrichment Analyses

Enrichment analyses were performed to identify biological functions associated with the plasma protein mortality signatures. The 9703 aptamers were first mapped to Entrez Gene identifiers using the *org.Hs.eg.db* annotation database. When multiple entries mapped to the same gene, the target with the largest absolute *z*‐statistic (logHR/SE) was retained. This resulted in 8700 unique genes, which were ranked in descending order according to their *z*‐statistics from the age‐ and sex‐adjusted Cox models and used as input for pre‐ranked gene set enrichment analysis (GSEA). GSEA was performed using the *clusterProfiler* package (version 4.14.6) to assess enrichment across Gene Ontology Biological Process, Cellular Component and Molecular Function categories using *gseGO* (Xu et al. 2024). Reactome pathway analysis was also conducted using *ReactomePA* (version 1.50.0) (Yu and He 2015). Gene sets were restricted to 10–500 genes, and significance was assessed using Benjamini‐Hochberg FDR correction (*p*_FDR_ < 0.05). For visualisation, the top 10 enriched terms were selected based on a composite ranking score, defined as the absolute normalised enrichment score (NES) multiplied by −log10(*p*_FDR_), to prioritise terms with both strong effect size and statistical significance.

## 3 Results

### 3.1 Characterising Proteomic Organ Ageing Signatures in the LBC1936

We predicted proteomic ages for 11 major organs in LBC1936 wave 2 participants using the LASSO models developed by Oh et al. (2023) (Figure S1A). Organ age gaps were calculated as the residuals from linear regressions of predicted organ age on chronological age and were standardised to have zero mean and unit variance (Figure S1B). These quantify the relative ageing status of each organ, with positive values indicating accelerated organ ageing and negative values indicating decelerated organ ageing relative to the cohort average. Despite individuals being of similar chronological age, we observed substantial variation in the biological ages of different organs both between and within individuals, with some organs appearing biologically older or younger than expected. We then examined the extent to which each organ age gap captured unique information. If organs aged at different rates, then the age gap of one organ should be independent from the age gap of another in the same individual. Consistent with previous findings, organ age gaps were only mildly correlated in the LBC1936 (mean pairwise Pearson *r* = 0.32; Figure S1C), closely matching the estimate reported in the original study (*r* = 0.29; Oh et al. 2023). We next characterised the prevalence and distribution of extreme organ ageing profiles. Extreme agers were defined as individuals with an organ age gap exceeding ±2 standard deviations from the population mean for at least one organ. Although one might expect extreme ageing in one organ to co‐occur with extreme ageing in others, extreme ageing most commonly affected a single organ rather than multiple organs simultaneously in the same individual. Specifically, most participants (70.9%, *N* = 563) exhibited no extreme organ ageing; 19% (*N* = 151) showed extreme ageing in one organ, 6% (*N* = 48) in two organs, 1.6% (*N* = 13) in three organs, 0.8% (*N* = 6) in four organs and 1.6% (*N* = 13) in five or more organs (multi‐organ agers) (Figure S1D). These proportions closely resemble those reported in the original study, in which 18.4% of individuals exhibited highly organ‐specific extreme ageing signatures and 1.7% showed extreme ageing in at least five organs (Oh et al. 2023).

### 3.2 Epigenetic, Neuroimaging and Functional Ageing Biomarkers Outperform Proteomic Organ Clocks in Associations With All‐Cause Mortality

We benchmarked 11 proteomic organ clocks against a broad panel of multimodal ageing biomarkers, all measured at ~73 years, for associations with all‐cause mortality in the LBC1936. For each ageing biomarker, we fitted three Cox proportional hazards models with incremental adjustment for (1) chronological age and sex, (2) lifestyle risk factors and (3) seven medical conditions. Results based on the maximum available sample size (*N* = 656–861) for each biomarker and on the subset of individuals with complete data across all biomarkers (*N* = 460) were nearly identical (Pearson *r* of log hazard ratios = 0.99) (Figure S2); we therefore present the results on the largest available sample size to maximise statistical power.

All ageing biomarkers were significantly associated with mortality (HR_range_ = 1.11–1.62, *p*_FDR_ < 0.05) after adjustment for chronological age and sex, except leukocyte telomere length. Among the proteomic organ clocks, a 1‐SD increase in each organ age corresponded to a 16%–43% higher mortality hazard. The most pronounced effects were observed for accelerated ageing of the liver (HR = 1.43, 95% CI = 1.30–1.58, *p*_FDR_ = 7.08 × 10^−13^), immune system (HR = 1.42, 95% CI = 1.29–1.57, *p*_FDR_ = 1.58 × 10^−11^) and heart (HR = 1.38, 95% CI = 1.25–1.53, *p*_FDR_ = 2.28 × 10^−10^). The proteomic brain age gap (HR = 1.31, 95% CI = 1.19–1.44, *p*_FDR_ = 1.21 × 10^−7^) showed a similar hazard ratio to the MRI‐derived brain age gap (HR = 1.30, 95% CI = 1.15–1.46, *p*_FDR_ = 1.77 × 10^−5^) (Figure 1).

**FIGURE 1:**
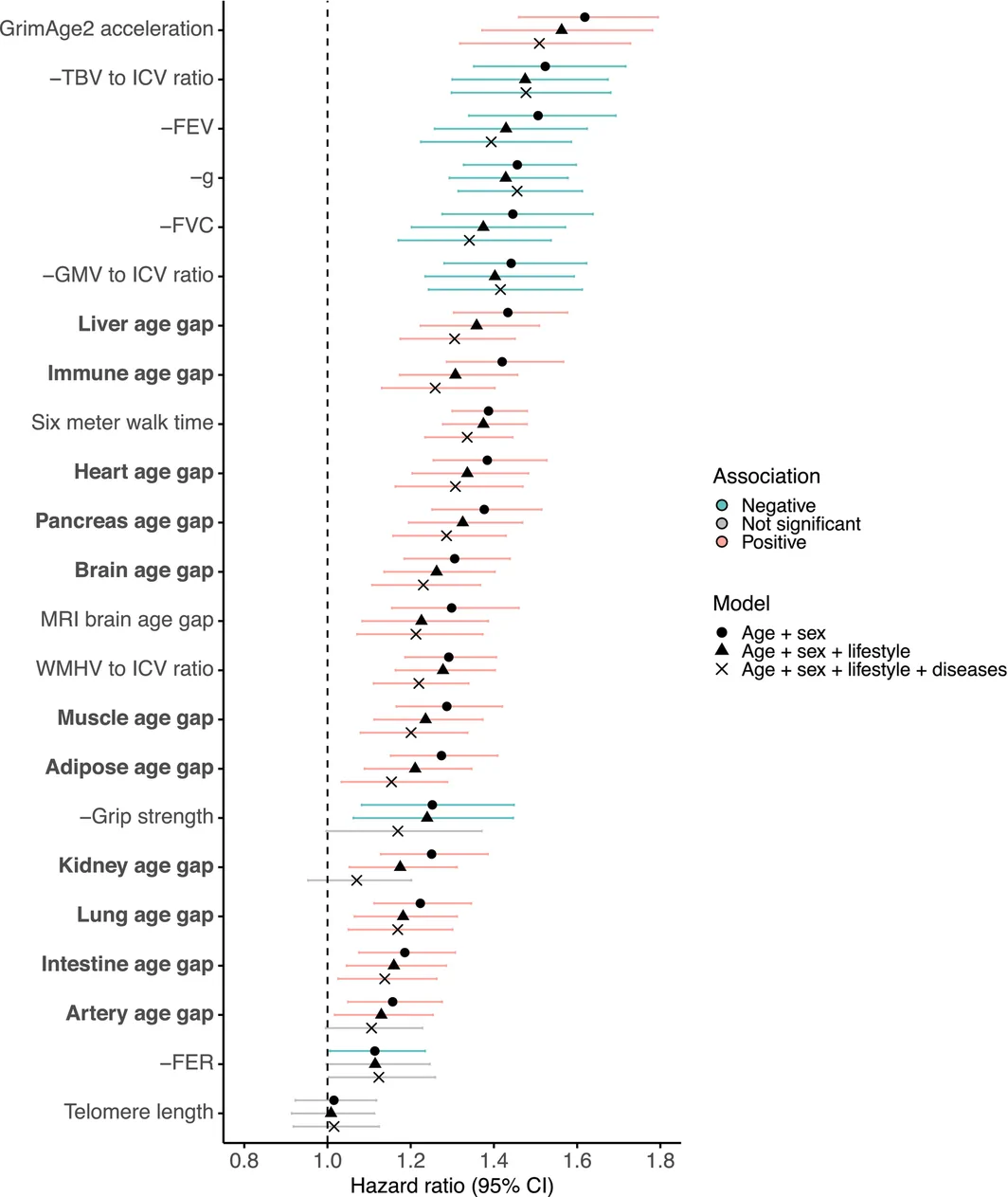
Associations of 23 ageing biomarkers with all‐cause mortality in the LBC1936. Associations of ageing biomarkers with 16‐year all‐cause mortality were estimated by fitting separate Cox proportional hazards models for each biomarker (*N* = 656–861 individuals; 326–444 deaths). Models were incrementally adjusted for age and sex (circle), lifestyle risk factors (smoking, alcohol consumption and BMI) (triangle) and seven major medical conditions (crosses). Biomarkers positively associated with mortality are shown in pink, and biomarkers negatively associated with mortality are highlighted in green. All negatively associated biomarkers are labelled with a minus sign preceding their name, and their reciprocal hazard ratios (1/HR) are plotted to facilitate comparison of effect sizes. The vertical dotted line indicates a hazard ratio of 1. Circles represent estimated hazard ratios, with error bars showing 95% confidence intervals. *p*‐values are adjusted using the Benjamini–Hochberg FDR correction, and non‐significant associations are shown in grey (*p*_FDR_ > 0.05). Proteomic organ clocks are indicated in bold. BMI, body mass index; FER, forced expiratory ratio; FEV, forced expiratory volume in 1 s; FVC, forced vital capacity; GMV/IVC, grey matter volume to intracranial volume ratio; MRI, magnetic resonance imaging; TBV/ICV, total brain volume to intracranial volume ratio; WMHV/ICV, white matter hyperintensity volume to intracranial volume ratio.

Several ageing biomarkers were more strongly associated with mortality than proteomic organ clocks. GrimAge2 acceleration demonstrated the largest overall association with mortality, with a 1‐SD increase conferring a 62% higher mortality hazard (HR = 1.62, 95% CI = 1.46–1.79, *p*_FDR_ = 6.78 × 10^−19^). Moreover, smaller TBV (reciprocal HR = 1.52, 95% CI = 1.35–1.72, *p*_FDR_ = 1.90 × 10^−11^) and GMV (reciprocal HR = 1.44, 95% CI = 1.28–1.62, *p*_FDR_ = 3.32 × 10^−9^), reduced respiratory function (FEV: reciprocal HR = 1.51, 95% CI = 1.34–1.69, *p*_FDR_ = 2.11 × 10^−11^; FVC: reciprocal HR = 1.45, 95% CI = 1.28–1.64, *p*_FDR_ = 1.41 × 10^−8^) and poorer cognitive performance (reciprocal HR = 1.46, 95% CI = 1.33–1.60, *p*_FDR_ = 1.39 × 10^−14^) were each associated with a 44%–52% higher mortality risk per 1‐SD decrease in their levels, surpassing all proteomic organ clocks in effect size. Domain‐specific trends revealed that GrimAge2 acceleration was the strongest predictor among molecular biomarkers, TBV among neuroimaging markers and FEV among physical function measures. Overall, adjustment for lifestyle factors, and subsequently for medical conditions, led to a small gradual attenuation of effect sizes for most biomarkers. FER became non‐significant after adjustment for lifestyle factors, while the proteomic artery and kidney clocks and grip strength became non‐significant after additional adjustment for disease status (Figure 1).

Detailed results, including hazard ratios, confidence intervals, *p*‐values, sample sizes and proportional hazards (PH) assumption violations, are provided in Table S2. For ageing biomarkers that violated the PH assumption, plots of hazard ratios per year of follow‐up are included in Figure S5 to demonstrate that hazard ratios remain largely stable, including instances where the assumptions are met, apart from the first few years when the incidence of death was low.

### 3.3 Neuroimaging, Cognitive and Physical Function Biomarkers of Ageing Independently Associate With All‐Cause Mortality

We next assessed whether the 23 ageing biomarkers captured overlapping or distinct aspects of ageing by calculating pairwise Pearson's correlations. Overall, most biomarkers showed low‐to‐moderate correlations, indicating that although they capture some redundant information, they mostly reflect unique aspects of ageing. GrimAge2, a composite epigenetic biomarker integrating multiple biological signals and smoking exposure, was correlated with most other ageing biomarkers, including all proteomic organ clocks (mean |*r*| = 0.20). Walking time was also correlated with multiple ageing biomarkers (mean |*r*| = 0.14), consistent with its role as an integrative measure of physical function that reflects the capacity of multiple physiological systems. As expected, the strongest correlations were observed between biomarkers within the same domain. Proteomic organ ages were mostly correlated with each other (*r*_range_ = 0.12–0.57), while displaying no or weak relationships with other ageing biomarkers, like neuroimaging (|*r*|_range_ = 0.11–0.16) and functional measures (|*r*|_range_ = 0.11–0.18). Similarly, older MRI‐derived brain age was most strongly associated with smaller MRI‐derived TBV (*r* = −0.47) and GMV (*r* = −0.43) but showed no correlations with proteomic organ clocks and most physical function measures. Nevertheless, several cross‐domain associations were observed between biologically related measures. For example, higher cognitive function was associated with greater TBV (*r* = 0.24), greater GMV (*r* = 0.20) and lower cerebrovascular damage (*r* = −0.17) (Figure S3).

As many biomarkers were correlated with one another, we next asked which biomarkers provided independent information on mortality risk after accounting for shared variance between measures. We therefore fitted a multivariable Cox proportional hazards model including all biomarkers simultaneously, with adjustment for chronological age, sex and lifestyle risk factors, among the 460 LBC1936 participants with complete biomarker data (Table S3). Biomarkers exhibiting strong collinearity (Pearson's *r* > 0.7, P_FDR_ < 0.05) were excluded, resulting in 21 biomarkers being included in the final model. Only four biomarkers—TBV, WMH volume, *g* and walk time—retained significant independent associations with mortality. The loss of significance for several biomarkers in the multivariable model reflects their correlation with other ageing measures, such that they provided limited additional information on mortality risk beyond that captured by these four core measures.

To further quantify the relative contribution of different biomarker domains to mortality risk, we compared Nagelkerke pseudo‐*R* ^2^ values from Cox models incorporating each domain separately. Age and sex accounted for 2% of the variance in mortality risk, with lifestyle factors contributing an additional 4%. GrimAge2 accounted for a further 6% beyond demographic and lifestyle factors, whereas proteomic organ clocks and physical function measures accounted for 8% and 9%, respectively. Brain‐related measures, including neuroimaging and cognitive function, accounted for around twice as much variance (16%). The combination of TBV, WMH volume, g and walking time—the biomarkers that retained independent associations in the multivariable model—accounted for 19% of the variance in mortality risk. Including all 21 biomarkers increased the variance accounted for to 23%, indicating that the additional biomarkers provided only limited incremental information beyond that captured by these four core measures (Figure S4).

### 3.4 Plasma Proteomic Signatures and Associated Biological Functions of All‐Cause Mortality

To identify proteomic biomarker candidates for assessing mortality risk and to characterise their underlying biological functions, we conducted a large‐scale survival analysis on 9703 SomaScan‐measured plasma protein targets in the LBC1936 (*N* = 789). Chronological age‐ and sex‐adjusted Cox proportional hazards regression identified 368 unique proteins significantly associated with mortality (*p*_FDR_ < 0.05), with 286 being associated with higher mortality risk and 82 with lower risk. After additional adjustment for lifestyle risk factors, including kidney function, 202 protein‐mortality associations remained significant. These were further reduced to 117 associations after adjusting for seven medical conditions (Figure 2A). In the age‐ and sex‐adjusted model, the strongest positive associations with mortality were observed for GDF15 (growth differentiation factor 15, HR = 1.56, 95% CI = 1.42–1.72, *p*_FDR_ = 3.66 × 10^−15^), WFDC2 (WAP four‐disulphide core domain protein 2, HR = 1.47, 95% CI = 1.33–1.62, *p*_FDR_ = 1.98 × 10^−10^), and TIMP1 (Tissue Inhibitor of Metalloproteinases 1, HR = 1.45, 95% CI = 1.29–1.62, *p*_FDR_ = 6.49 × 10^−8^). Conversely, NPS (neuropeptide S, reciprocal HR = 1.42, 95% CI = 1.29–1.56, *p*_FDR_ = 7.84 × 10^−10^), BAGE3 (B melanoma antigen 3, reciprocal HR = 1.41, 95% CI = 1.28–1.55, *p*_FDR_ = 2.28 × 10^−9^) and ABCC6 (ATP‐Binding Cassette Subfamily C Member 6, reciprocal HR = 1.41, 95% CI = 1.17–1.68, *p*_FDR_ = 9.76 × 10^−3^) showed the strongest negative associations with mortality. Additional adjustment for lifestyle risk factors and medical conditions generally resulted in modest attenuation of effect sizes for 17 of the 20 top proteins (Figure 2B). Overall, 16 of the top 20 proteins were shared between the demographic and fully adjusted Cox models, with ABCC6, PRSS8, RNASE1 and CEMP1 identified only in the demographic model, and CYP1A1, PXDN, FGL1 and IGFBP2 identified only in the fully adjusted model. Overall, GDF15, WFDC2 and TIMP1 (HR_range_ = 1.45–1.56) showed stronger associations with mortality than proteomic organ clocks (HR_range_ = 1.16–1.43). Additionally, plasma GDF15 (HR = 1.56) emerged as the second strongest mortality risk predictor following GrimAge2 (HR = 1.62). In total, 3 of the 9 plasma proteins for which DNAm surrogates are included in GrimAge2 (GDF15, CST3 and TIMP1) were among the top 20 mortality‐associated proteins identified in this study. Hazard ratios, *p*‐values, sample sizes and PH assumption violations for all proteins are reported in Table S4, and yearly hazard ratios for proteins violating the PH assumption are presented in Figure S6.

**FIGURE 2:**
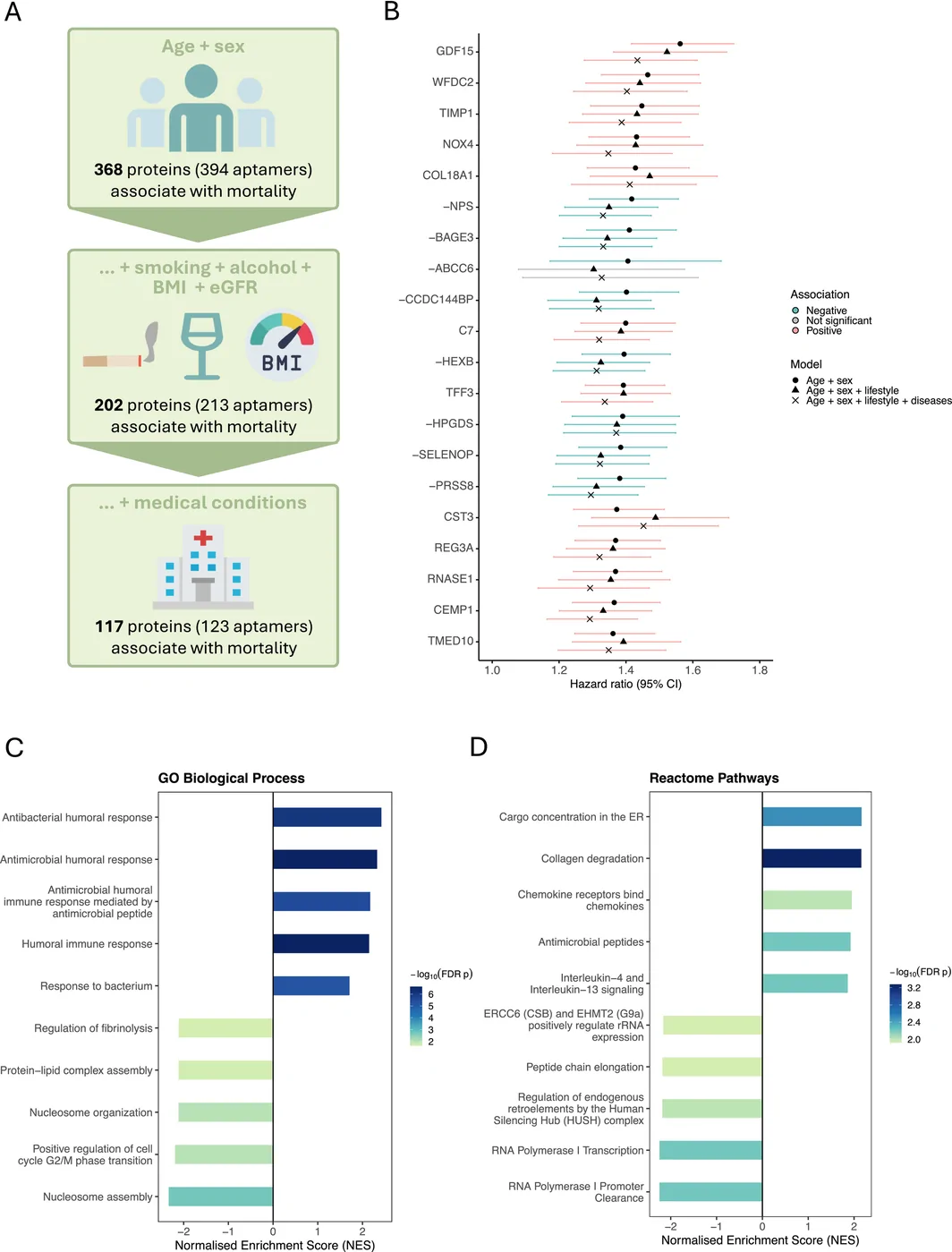
Plasma proteins and biological functions associated with all‐cause mortality in the LBC1936. (A) Cox models were applied to 9703 SomaScan plasma protein targets to test associations with 16‐year all‐cause mortality, with incremental adjustment for age and sex, lifestyle factors (smoking, alcohol and BMI) and seven medical conditions. The number of significant aptamers and unique proteins after Benjamini–Hochberg FDR correction is shown. (B) Top 20 plasma proteins associated with all‐cause mortality after adjustment for age and sex (*N* = 789 individuals; 404 deaths). Hazard ratios and 95% confidence intervals are plotted with incremental adjustment for age and sex (circles), lifestyle factors (triangles) and medical conditions (crosses). Positive associations are shown in pink and negative associations in green. Negatively associated proteins are labelled with a minus sign preceding their name, and their reciprocal hazard ratios (1/HR) are plotted. *p*‐values are adjusted using the Benjamini–Hochberg FDR correction, and non‐significant associations are shown in grey (*p*_FDR_ > 0.05). (C‐D) Top five positively and negatively enriched Gene Ontology biological processes (C) and Reactome pathways (D) among mortality‐associated proteins identified by Gene Set Enrichment Analysis. The normalised enrichment score (NES) shows the magnitude and direction of pathway enrichment while accounting for differences in gene set size. Top terms were selected using a composite ranking score calculated as |NES| × −log10(P_FDR_). The colour scale indicates statistical significance on the −log10(*p*_FDR_) scale.

We further characterised the biological functions enriched among proteomic mortality signals using GSEA, revealing a marked functional dichotomy between proteins associated with increased versus decreased mortality risk. Proteins with higher plasma levels associated with increased mortality risk were predominantly enriched for immune‐related GO biological processes, particularly antimicrobial humoral immune responses (Figure 2C). At the molecular function level, these proteins showed enrichment for chemokine activity, cytokine activity, receptor ligand binding and signalling receptor activity. Consistently, Reactome pathway analysis identified enrichment of chemokine signalling, antimicrobial peptide pathways and interleukin‐4 and interleukin‐13 signalling, highlighting a prominent association of immune processes with higher mortality risk (Figure 2D).

In contrast, proteins associated with lower mortality risk were enriched for processes related to genomic integrity, including nucleosome assembly and organisation, and regulation of G2/M phase cell cycle transition (Figure 2C). Molecular function enrichment included structural constituents of chromatin and damaged DNA binding, while Reactome analysis highlighted additional pathways involved in ribosome biogenesis, protein synthesis, and regulation of endogenous retroelements (Figure 2D). Together, these findings suggest that elevated mortality risk is characterised by increased circulating levels of proteins involved in immune responses, whereas reduced mortality risk is associated with higher levels of proteins supporting genomic stability and core cellular maintenance processes.

To account for the high dimensionality and multicollinearity of plasma proteomic data, we fitted an elastic net‐penalised Cox proportional hazards regression model simultaneously to 9703 SomaScan 11K plasma protein targets measured in LBC1936 wave 2 participants (5‐fold cross‐validation; *α* = 0.5; λ_min_ = 0.16). In this model, each protein's coefficient represents the log hazard ratio for a 1‐SD increase in its plasma level, with positive values indicating higher mortality risk and negative values indicating lower risk. The model retained 37 proteins with non‐zero coefficients; GDF15 (coefficient = 0.18, HR ≈1.20) had the largest positive coefficient, while HPGDS (coefficient = −0.09, HR ≈0.91) had the largest negative coefficient. In total, 33 of the 37 elastic net proteins (89.2%) overlapped with those identified by the sex‐ and age‐adjusted mortality Cox models, indicating strong concordance between the two approaches. Estimated elastic net model protein coefficients are plotted in Figure 3 and reported in Table S5.

**FIGURE 3:**
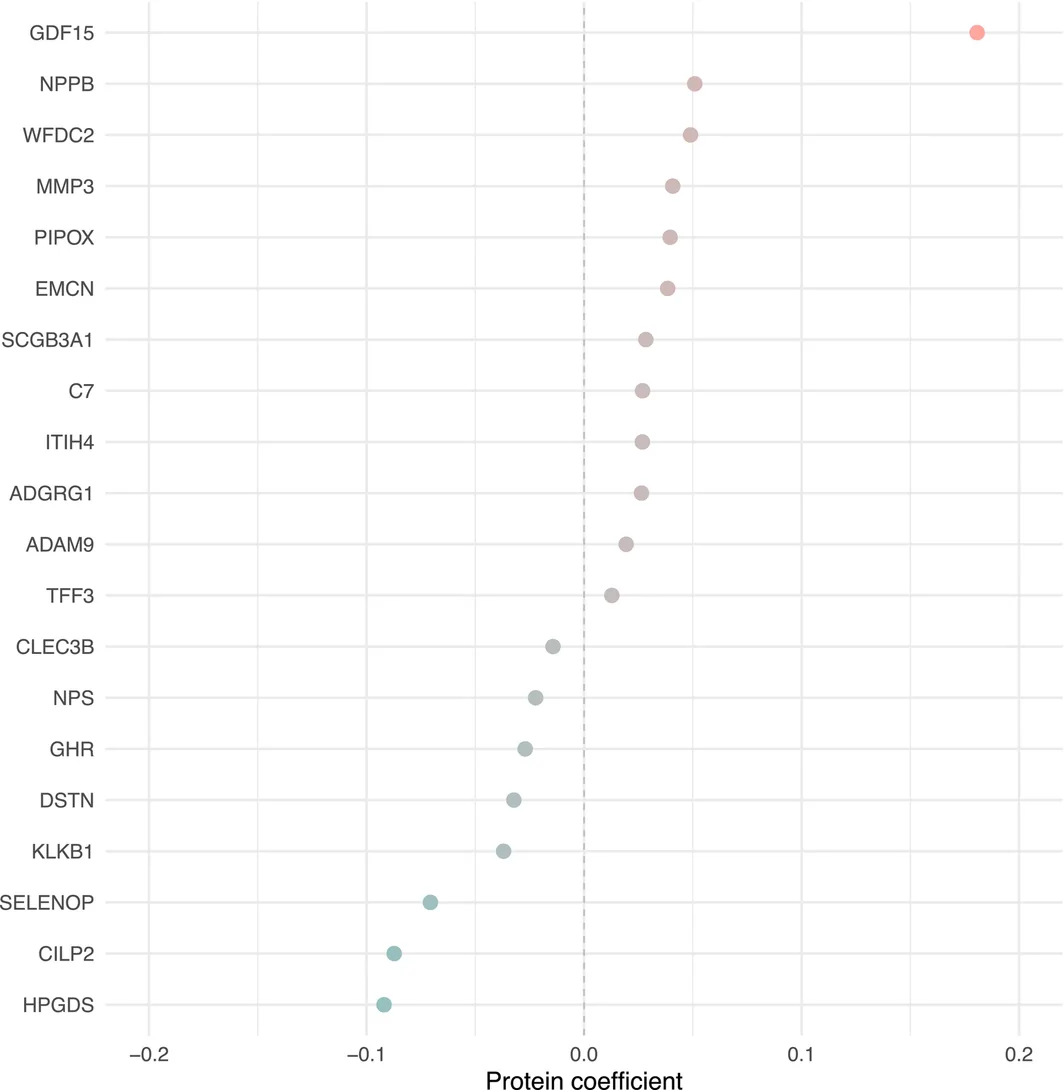
Top 20 plasma protein predictors of all‐cause mortality in the LBC1936. The plot shows the 20 SomaScan 11k plasma proteins with the largest coefficients from a total of 37 proteins selected to predict all‐cause mortality using elastic net‐penalised Cox regression (*N* = 789 individuals; 404 deaths). The mixing parameter (*α*) was set to 0.5, and the penalty parameter (λ) was chosen as 0.16 based on the λ_min_ from 5‐fold cross‐validation. Proteins with positive coefficients, indicating higher mortality risk, are shown in pink, while proteins with negative coefficients, indicating lower mortality risk, are shown in green.

## 4 Discussion

This study benchmarked 11 plasma proteomic organ clocks against other multimodal ageing biomarkers in relation to all‐cause mortality over 16 years of follow‐up in the LBC1936. Accelerated organ ageing conferred a 16%–43% higher mortality risk per 1‐SD increase, with the liver, immune system and heart showing the largest effects. However, greater GrimAge2 acceleration, smaller MRI‐derived brain volumes (TBV and GMV), reduced respiratory function (FEV and FVC) and lower cognitive function exhibited larger hazard ratios than any proteomic organ clock, increasing mortality risk by 44%–62%. Additionally, when all ageing biomarkers were modelled simultaneously, only TBV, WMH volume, *g* and walk time remained significantly associated with mortality and explained 19% of variance in mortality risk. Survival analyses of 9703 plasma protein targets identified 368 candidates associated with mortality and revealed that proteins associated with increased mortality risk were predominantly involved in immune functions, whereas protective proteins were enriched for roles in genomic integrity and cellular maintenance. GDF15, WFDC2 and TIMP1 showed the strongest positive associations with mortality risk, increasing it by 45%–56% per 1‐SD increase in plasma levels. In contrast, NPS, BAGE3 and ABCC6 were the top negatively associated proteins, increasing mortality risk by 41%–42% per 1‐SD decrease in plasma levels. A penalised regression model with all proteins as inputs selected 37 candidates in a parsimonious solution, with GDF15, Natriuretic Peptide B (NPPB) and WFDC2 among the most influential predictors. Overall, this study comprehensively benchmarks proteomic organ clocks against other multimodal ageing biomarkers and uncovers new plasma proteomic signatures and biological pathways associated with mortality.

### 4.1 Proteomic Organ Clocks Associate With Mortality Risk but Are Outperformed by Epigenetic, Neuroimaging and Functional Biomarkers of Ageing

Our study confirms that accelerated proteomic organ ageing is associated with increased mortality risk in an independent longitudinal cohort of older adults. Our findings align with the original study, which examined associations between the 11 organ clocks and 15‐year all‐cause mortality risk in the LonGenity cohort (*N* = 864) using age‐ and sex‐adjusted Cox models (Oh et al. 2023). The LonGenity study reported a 17%–53% increase in mortality risk per 1‐SD increase in organ age, compared with a 16%–43% increase observed in our study. Furthermore, we demonstrate that proteomic organ clocks perform modestly relative to other ageing biomarkers, including GrimAge2, MRI‐derived brain volume measures, respiratory function and cognition.

GrimAge2 emerged as the strongest biomarker of mortality risk in the LBC1936. Consistently, GrimAge2 has previously been identified as the strongest predictor of 10‐year all‐cause mortality among 14 widely used epigenetic clocks in the Generation Scotland (GS) cohort (*N* = 18,859) (Mavrommatis et al. 2025). In GS, a 1‐SD increase in GrimAge2 acceleration was associated with a 54% higher mortality risk, which is broadly comparable to the 62% increase observed in the LBC1936. Another study benchmarking 11 epigenetic clocks in the National Health and Nutrition Examination Survey (NHANES) cohort (*N* = 1942) also identified GrimAge2 to be the most effective epigenetic biomarker for mortality risk prediction (Zhu et al. 2025). Its superior performance likely reflects its ability as a composite biomarker to capture multiple biological ageing processes (including cellular senescence, inflammation, vascular and renal dysfunction and metabolic alterations) and cumulative smoking exposure, as well as its direct training on mortality outcomes.

MRI‐derived TBV was the second strongest ageing biomarker associated with mortality risk. This finding aligns with previous studies reporting associations between several indices of brain atrophy and reduced survival (Van Elderen et al. 2016; Olesen et al. 2011; Ikram et al. 2009; Kuller et al. 2007; Staff et al. 2010). In the population‐based AGES‐Reykjavik cohort (*N* = 3543), which was free of prevalent stroke and dementia and of similar age to the LBC1936, lower TBV was associated with a 52% higher risk of all‐cause mortality after adjustment for age and sex (Van Elderen et al. 2016), an effect size identical to that observed in the LBC1936. Several factors may explain why brain atrophy is associated with reduced survival. First, the brain regulates essential physiological functions, including blood pressure, thermoregulation, appetite, energy balance, fluid and electrolyte homeostasis, as well as motor, behavioural and cognitive processes critical for survival (Tabarean et al. 2010; McKinley et al. 1999; Skoog et al. 1998; Guo et al. 2001). Second, brain atrophy is related to midlife risk factors such as hypertension and obesity, supporting its role as an indicator of cumulative health burden (Gustafson et al. 2004; Swan et al. 1998). However, its strong association with mortality persisted even after adjustment for such risk factors in the LBC1936, suggesting that it captures aspects of biological vulnerability beyond these established vascular and metabolic risk factors. Third, reduced brain volume may represent an early marker of neurodegeneration and dementia, which is the strongest disease predictor of survival in old age (Wetterberg et al. 2021; Tschanz et al. 2004). Its ability to capture neurodegenerative processes before the onset of cognitive impairment or clinical diagnosis may make it a more sensitive predictor of survival (Olesen et al. 2011; Tondelli et al. 2012; Fletcher et al. 2018; Sluimer et al. 2008; Mungas et al. 2005). Collectively, these findings underscore the importance of brain health in predicting survival at old age.

Respiratory function (FEV) showed a similar association with mortality to TBV, with a 51% higher mortality risk per 1‐SD decrease in function. Several studies have reported significant associations between reduced respiratory function and increased mortality risk (Magnussen et al. 2017; Ching et al. 2019; Costanzo et al. 2021; Court et al. 2022). The Gutenberg Health Study (*N* = 12,703), which included younger individuals aged 35–74 years, found that a 1‐SD reduction in respiratory function was associated with a 28% higher mortality risk after adjustment for age, sex, BMI, diabetes, smoking, dyslipidemia, hypertension and heart failure (Magnussen et al. 2017). Similarly, the Moli‐sani study (*N* = 14,503; mean age = 53 years) reported a 24% higher risk of death after adjustment for age and sex (Costanzo et al. 2021). The larger effect size observed in the LBC1936 may at least partly reflect the older age of this cohort. Lungs undergo substantial anatomical, physiological and immunological changes with age, and the decline in FEV with ageing seems to be nonlinear, with an accelerated rate of decline seen after age 70 (Sharma and Goodwin 2006). Respiratory function is an important indicator of survival for several reasons. First, respiratory function represents an integrated measure of multi‐system physiological capacity, as it depends on the coordinated function of the respiratory, cardiovascular, neuromuscular and musculoskeletal systems. Second, reduced lung function and impaired oxygen delivery can have widespread systemic consequences, with the cardiovascular and nervous systems being particularly vulnerable. For example, reduced respiratory function increases cardiovascular strain and is an independent and causal risk factor for cardiovascular disease (Duong et al. 2019; Higbee et al. 2022). Additionally, lung function is highly sensitive to cumulative lifestyle and environmental exposures (Pouptsis et al. 2025), which explains the substantial attenuation in its association with mortality after adjustment for lifestyle factors in the LBC1936. A study investigating the contribution of socioeconomic factors, health behaviours, cardiovascular risk factors, inflammatory markers and chronic diseases to the association between lung function and mortality found that, after accounting for age, sex and smoking, inflammatory markers explained the largest proportion of the association (21% reduction in the association), followed by chronic diseases (12%) and lifestyle risk factors (10%) (Sabia et al. 2010). However, the directional relationship between lung function and inflammation remains debated. Overall, respiratory function is a strong biomarker of survival at old age, reflecting multi‐system physiological reserve and the burden of environmental and lifestyle exposures, while offering a more practical and affordable alternative to MRI‐derived brain biomarkers.

### 4.2 Brain Atrophy, Cerebrovascular Damage, Poor Cognition and Slower Walking Show the Strongest Independent Associations With Mortality Risk

Smaller brain volume, greater cerebrovascular damage, poorer cognition and slower walking speed were the only biomarkers that remained independently associated with mortality after accounting for all other ageing biomarkers. When correlated predictors are included in the same model, effect sizes can attenuate due to shared variance and collinearity, so only biomarkers with sufficiently independent information remain significant. As a result, strong univariable predictors such as GrimAge2 appear non‐significant in the multivariable model not because they lack biological relevance, but because much of their prognostic signal overlaps with other correlated ageing biomarkers. Together, the four independently associated measures accounted for the greatest proportion of variance in mortality risk (approximately one‐fifth), suggesting that combining complementary measures across different modalities may be most informative for mortality risk assessment.

### 4.3 Proteins Associated With Higher Mortality Risk Are Enriched for Immune Functions, Whereas Those Associated With Lower Risk Relate to Genomic Stability and Cellular Maintenance

Our survival analysis of 9703 plasma protein targets identified 368 mortality‐associated candidates, providing a resource for prioritising biomarkers for mortality risk stratification, mechanistic studies and therapeutic development. To our knowledge, this is among the first studies to characterise proteomic signatures of mortality at this scale, with only one other SomaScan 11K plasma protein analysis currently available as a preprint from the MRC National Survey of Health and Development (NSHD) cohort (*N* = 1803; mean age = 63 years) (Groves et al. 2025). Although detailed NSHD results have not yet been publicly reported, we replicated six of its top 15 mortality‐associated proteins (GDF15, WFDC2, TIMP1, CST3, RNASE1 and SELENOP). We also confirmed significant and directionally consistent associations for MED9, ANTXR2 and COL6A3, although these ranked lower in the LBC1936.

We further functionally characterised proteomic mortality signals and revealed striking functional dichotomy, with high‐risk proteins enriched for immune functions and low‐risk proteins involved in genomic stability and cellular maintenance. Immunosenescence, the age‐related decline and remodelling of innate and adaptive immunity accompanied by chronic low‐grade inflammation, is a key hallmark of ageing (López‐Otín et al. 2023). This process increases susceptibility to infections, reduces vaccine efficacy, impairs cancer surveillance, promotes tissue damage and enhances autoimmunity, collectively elevating the risk of infections, age‐related diseases and ultimately mortality (Liu et al. 2023; Goyani et al. 2024; Lee et al. 2022). GDF15, a stress‐ and inflammation‐induced cytokine, emerged as the strongest mortality‐associated protein. This protein is a component of the senescence‐associated secretory phenotype, and its circulating levels increase with age and promote immunosenescence through chronic immunosuppression (Salminen 2025). Circulating GDF15 is also elevated across multiple age‐related diseases (including cardiovascular disease, neurodegeneration and cancer) and consistently associates with all‐cause mortality across cohorts (Bao et al. 2021; Conte et al. 2022; Gadd et al. 2024; Lajer et al. 2010; Lyu et al. 2024). Beta‐2 microglobulin (B2M) is another widely recognised systemic pro‐ageing factor that actively drives inflammaging and cognitive decline, and its circulating levels increase with age and are associated with survival (Hilt et al. 2019; Huang et al. 2022; Shinkai 2008). Furthermore, B2M deficiency rescues cognitive decline and enhances neurogenesis in aged mice, supporting its potential as a therapeutic target for healthy ageing (Smith et al. 2015). Elevated chemokines (e.g., CXCL8, CCL3, CCL14, CCL15 and CCL28) were also associated with higher mortality risk, consistent with their central role in inflammaging. Notably, chemokine receptor antagonists and anti‐chemokines are being investigated as interventions to promote healthy ageing and extend lifespan (Chaudhary et al. 2025). We also identified less well‐characterised candidates, including regenerating islet‐derived (REG) proteins, which are secreted C‐type lectins with antimicrobial and pro‐regenerative functions that are upregulated in response to injury, infection or inflammation, and members of the whey acidic protein four‐disulfide core (WFDC) family with antimicrobial, immunomodulatory and tissue remodelling functions (Chen et al. 2019; Wen et al. 2024).

In contrast, proteins associated with lower mortality risk were strongly enriched for processes involved in genomic stability, including nucleosome assembly and organisation, G2/M cell‐cycle checkpoint regulation and DNA damage binding. Genomic instability is a well‐established hallmark of ageing, as progressive accumulation of DNA damage and declining repair capacity compromise tissue integrity and increase susceptibility to age‐related diseases, including cancer (López‐Otín et al. 2023). Defects in DNA repair mechanisms accelerate ageing in animal models and contribute to premature ageing syndromes in humans (Perez et al. 2024; Hennekam 2020). Conversely, overexpression of enzymes involved in cell cycle checkpoints and DNA repair has been shown to extend lifespan in animal models (North et al. 2014; Tian et al. 2019; Gordon et al. 2014). In this study, we found that higher plasma levels of proteins involved in maintaining genomic integrity, including Sirtuin‐2 (SIRT2) and Caspase‐2 (CASP2), were associated with 18% and 32% lower mortality risk, respectively. SIRT2 is critical for genome maintenance and tumour suppression by regulating a network of DNA damage response proteins via deacetylation (Zhang et al. 2013; Zhang et al. 2016; Kim et al. 2011; Zhang et al. 2016). Overexpression of SIRT2 in mice also increases lifespan by inducing the checkpoint kinase BubR1 (North et al. 2014). CASP2 stabilises p53 in response to cellular stress and DNA damage to induce cell cycle arrest and apoptosis, preventing the accumulation of damaged cells and suppressing tumorigenesis (Puccini et al. 2013; Lim et al. 2021). Furthermore, CASP2‐deficient mice exhibit signs of premature ageing, including shortened lifespan, increased bone loss, reduced body fat and elevated oxidative stress (Shalini et al. 2012; Zhang et al. 2007). Beyond genome maintenance, we also identified a novel association between higher circulating Neuropeptide S levels and a 42% lower mortality risk. Although not previously linked to lifespan, Neuropeptide S has been shown to confer neuroprotection, enhance memory and reduce anxiety, and is therefore being investigated as a therapeutic target for anxiety disorders and neurodegenerative conditions (Pape et al. 2010; Shirsath et al. 2024; Zhao et al. 2019). It also suppresses oxidative stress and food intake, which may be relevant to healthy ageing (Shirsath et al. 2024). Further mechanistic studies are required to determine whether Neuropeptide S has causal effects on lifespan and whether it represents a potential anti‐ageing target.

### 4.4 Limitations

Although this study has notable strengths, including a broad range of ageing biomarkers, large‐scale unbiased proteomic profiling and direct effect‐size comparisons within a single population, several limitations should be considered. First, LBC1936 consists of relatively healthy, ethnically homogeneous Scottish individuals of similar chronological age, and validation of our findings in more diverse populations is needed. Second, our analyses relied on baseline biomarker values and did not incorporate longitudinal trajectories, which may provide greater predictive value than single time‐point measurements. Future analyses will consider protein trajectories with health and survival. Third, due to sample size, we examined all‐cause rather than cause‐specific mortality, which may be more informative for organ age biomarkers. Fourth, we used a single proteomics platform (SomaScan 11K) and were therefore dependent on the reliability of those protein measurements. Finally, our results are observational and do not establish causality. Further functional and mechanistic studies will be required to determine whether specific proteins play causal roles in ageing and lifespan.

## 5 Conclusion

This study benchmarked plasma proteomic organ clocks against a broad panel of multimodal ageing biomarkers in relation to all‐cause mortality. Although accelerated organ ageing was associated with increased mortality risk, other biomarkers, including GrimAge2, neuroimaging, cognitive and respiratory function measures, showed stronger associations. Whether organ clocks provide additional value for cause‐specific mortality remains unclear. After accounting for shared variance among biomarkers, only greater brain atrophy, increased cerebrovascular damage, poorer cognition and slower walking remained independently associated with mortality, highlighting the importance of integrating complementary cross‐modal measures for mortality risk assessment. GrimAge2 showed the strongest association with mortality, suggesting that integrative biomarkers capturing multiple biological ageing signals and environmental exposures, and trained directly on mortality outcomes, offer optimal performance. Furthermore, large‐scale plasma proteomic analyses identified both established and novel protein candidates and biological pathways underlying mortality risk, revealing enrichment of immune‐related processes among high‐risk proteins and genomic maintenance pathways among low‐risk proteins. These findings prioritise biologically plausible targets for mortality risk assessment, mechanistic validation and therapeutic development. Future studies should evaluate these biomarkers across diverse populations and age ranges, and experimentally validate candidate proteins to establish causality and therapeutic potential.

## Author Contributions

M.P., R.E.M. and S.E.H. designed the study. M.P. conducted all data analyses and wrote the original manuscript draft. I.M.E. performed quality control of the SomaScan 11K plasma proteomic data for LBC1936. P.R. and J.C. were responsible for data wrangling and quality assurance as well as for data collection, linkage and mortality follow‐up in the LBC1936. M.V.H. and J.M.W. contributed to the brain MRI methods section. All authors provided critical feedback, reviewed and edited the manuscript.

## Funding

The Lothian Birth Cohorts received joint funding from the BBSRC and ESRC (BB/W008793/1, to S.R.C., which supported S.E.H., J.C. and P.R.), the MRC (MR/R024065/1), Age UK (The Disconnected Mind), the Milton Damerel Trust and the University of Edinburgh. DNA methylation profiling was funded by the Centre for Cognitive Ageing and Cognitive Epidemiology (Pilot Fund Award), Age UK, the Wellcome Trust Institutional Strategic Support Fund, The University of Edinburgh and The University of Queensland. M.P. was supported by the Translational Neuroscience PhD Programme, funded by the Wellcome Trust (218493/Z/19/Z). S.R.C was supported by a Sir Henry Dale Fellowship, jointly funded by the Wellcome Trust and the Royal Society (221890/Z/20/Z). S.E.H., S.R.C and R.E.M. were additionally funded by the National Institutes of Health (NIH) research grant (U01AG083829), which also supported I.M.E., T.C.R., I.J.D., E.M.T.D., M.V.H., and by the BBSRC grant (UKRI/BB/C001941/1). J.M.W. was supported by the UK DRI, which is funded by the MRC, Alzheimer's Society and Alzheimer's Research UK, as well as the NIH. M.V.H. received funding from the Row Fogo Charitable Trust (BRO‐D.FID3668413, to J.M.W.). This work was also supported in part by the National Institute on Aging (NIA) Intramural Research Program (K.A.W.). The contributions of the NIH author(s) are considered Works of the United States Government. The findings and conclusions presented in this paper are those of the author(s) and do not necessarily reflect the views of the NIH or the U.S. Department of Health and Human Services.

## Ethics Statement

LBC1936 ethical approval was obtained from the Scotland A Research Ethics Committee (07/MRE00/58). All participants provided written informed consent.

## Conflicts of Interest

R.E.M. is an advisor to the Epigenetic Clock Development Foundation and Optima Partners Ltd. K.A.W. is an Associate Editor for Alzheimer's & Dementia: The Journal of the Alzheimer's Association, Alzheimer's & Dementia: Translational Research and Clinical Interventions (TRCI), and on the Editorial Board of Annals of Clinical and Translational Neurology. K.A.W. is on the Board of Directors of the National Academy of Neuropsychology. K.A.W. has given unpaid presentations and seminars on behalf of SomaLogic. K.A.W. is a co‐founder of Centia Bio. The work presented in this manuscript was conducted independently of Centia Bio and without financial support from the company. The remaining authors have no conflicts of interest to disclose.

## Supporting information


**Figure S1:** Characterisation of plasma proteomic organ ageing signatures in the LBC1936. (A) Predicted plasma proteomic ages for 11 organs in LBC1936 wave 2 participants (*N* = 795). Each point represents an individual, and the black dashed line indicates the mean organ age. (B) Density plots of organ age gaps, defined as standardised residuals (mean = 0, standard deviation = 1) from the linear regression of predicted organ age on chronological age. Positive values, indicating accelerated ageing, are shown in pink, and negative values, indicating slower ageing, are shown in green. (C) Pairwise Pearson correlation matrix of organ age gaps. Only significant correlations (*p*_FDR_ < 0.05) are shown. Positive correlations are shown in pink, and negative correlations in green. (D) Number and percentage of individuals with extreme ageing in one or more organs. Extreme ageing is defined as an organ age gap exceeding ±2 standard deviations from the population mean in at least one organ.
**Figure S2:** Comparison of all‐cause mortality associations for 23 ageing biomarkers using maximum versus common sample sizes. Associations between 23 ageing biomarkers measured at wave 2 and 16‐year all‐cause mortality were estimated using age‐ and sex‐adjusted Cox proportional hazards models in the LBC1936. Log hazard ratios derived from the maximum available sample for each biomarker (*N* = 656–861) are plotted against those obtained from the common subset with complete data across all biomarkers (*N* = 460).
**Figure S3:** Correlations among ageing biomarkers and modifiable lifestyle risk factors in the LBC1936. Pairwise Pearson correlations among 23 ageing biomarkers and modifiable lifestyle risk factors measured at wave 2 (*N* = 460). Red indicates positive correlations and green indicates negative correlations, with colour intensity reflecting correlation strength. Only significant associations are shown (*p*_FDR_ < 0.05).
**Figure S4:** Explanatory power for all‐cause mortality across different ageing biomarker domains. Nagelkerke's pseudo‐*R* ^2^ was calculated to assess the relative explanatory power of Cox proportional hazards models incorporating different combinations of predictors in the LBC1936. All biomarkers were measured at wave 2 (*N* = 460), and all‐cause mortality was assessed over an average of 16 years of follow‐up. Eight models were fitted with the following predictor sets: (1) demographics; (2) demographics + lifestyle; (3) demographics + lifestyle + GrimAge2; (4) demographics + lifestyle + organ clocks; (5) demographics + lifestyle + physical function; (6) demographics + lifestyle + brain measures; (7) demographics + lifestyle + four significant biomarkers; and (8) demographics + lifestyle +21 ageing biomarkers. Demographic factors included age and sex, and lifestyle factors included smoking, alcohol consumption, and body mass index (BMI). Organ clocks comprised the 11 plasma proteomic clocks. Physical function measures included grip strength, respiratory function and walking time. Brain measures included MRI‐derived total brain volume, white matter hyperintensity volume, brain age gap and general cognitive function. The four significant biomarkers were total brain volume, white matter hyperintensity volume, general cognitive function and walking time, as these remained independently associated with mortality after adjustment for all other ageing biomarkers.
**Figure S5:** Change in ageing biomarker associations with all‐cause mortality over 17 years of follow‐up in the LBC1936. Cox proportional hazards models were used to examine associations between individual ageing biomarkers measured at wave 2 and all‐cause mortality. Models were fitted annually across 17 years of follow‐up and adjusted for chronological age, sex, lifestyle risk factors and seven medical conditions. Forest plots show the seven ageing biomarkers that violated the proportional hazards assumption. Years with significant violations (Schoenfeld *p* < 0.05) are highlighted in red. Circles represent hazard ratios and error bars indicate 95% confidence intervals. The horizontal dotted line denotes a hazard ratio of 1.
**Figure S6:** Change in plasma protein associations with all‐cause mortality over 17 years of follow‐up in the LBC1936. Cox proportional hazards models were used to examine associations between individual SomaScan 11K plasma proteins measured at wave 2 and all‐cause mortality. Models were fitted annually across 17 years of follow‐up and adjusted for chronological age, sex, lifestyle risk factors and seven medical conditions. Forest plots show the 10 proteins among the top 20 mortality‐associated proteins that violated the proportional hazards assumption. Years with significant violations (Schoenfeld *p* < 0.05) are highlighted in red. Circles represent hazard ratios and error bars indicate 95% confidence intervals. The horizontal dotted line denotes a hazard ratio of 1.
**Table S1:** Quality control status of SomaScan 11K plasma proteins in the LBC1936. The 11,083 measured aptamers were classified as (1) passing quality control, (2) being non‐human targets, (3) likely to be affected by pre‐analytical variation, (4) having a high coefficient of variation, (5) having a high relative fluorescence unit signal or (6) having a signal‐to‐noise ratio < 1.
**Table S2:** Associations of individual ageing biomarkers with all‐cause mortality in the LBC1936. Cox proportional hazards regression results are presented for associations between individual biomarkers and 16‐year all‐cause mortality, with incremental adjustment for demographic variables, modifiable lifestyle risk factors and seven medical conditions.
**Table S3:** Associations of all ageing biomarkers with all‐cause mortality in the LBC1936. Cox proportional hazards regression results are presented for a model including all ageing biomarkers simultaneously and adjusted for demographic variables and modifiable lifestyle risk factors.
**Table S4:** Associations of individual plasma proteins with all‐cause mortality in the LBC1936. Cox proportional hazards regression results are presented for associations between individual plasma proteins and 16‐year all‐cause mortality, with incremental adjustment for demographic variables, modifiable lifestyle risk factors and seven medical conditions.
**Table S5:** Elastic net‐derived weights for the 37 plasma protein predictors of all‐cause mortality in the LBC1936.

## Data Availability

All data produced in the present study are reported in Tables S1–S5. The code used for analyses is openly accessible at the following GitHub repository: https://github.com/marioni‐group/Mortality_Biomarkers_LBC1936.git. The original data analysed in this study are not publicly available because they contain individual participant level information that would compromise participant consent. Data access instructions for the LBC are available at: https://www.ed.ac.uk/lothian‐birth‐cohorts/data‐access‐collaboration.
