# Supplementary material for: Multimodal Ageing Biomarkers and Plasma Proteomic Signatures Associated With All‐Cause Mortality
Source: Aging Cell. 2026 Oct 2;25(10):e70747. doi: 10.1111/acel.70747 (PMC13631562; doi:10.1111/acel.70747)
Supplement: Supplementary file 1 [file ACEL-25-e70747-s001.zip › FigureS5.pdf]

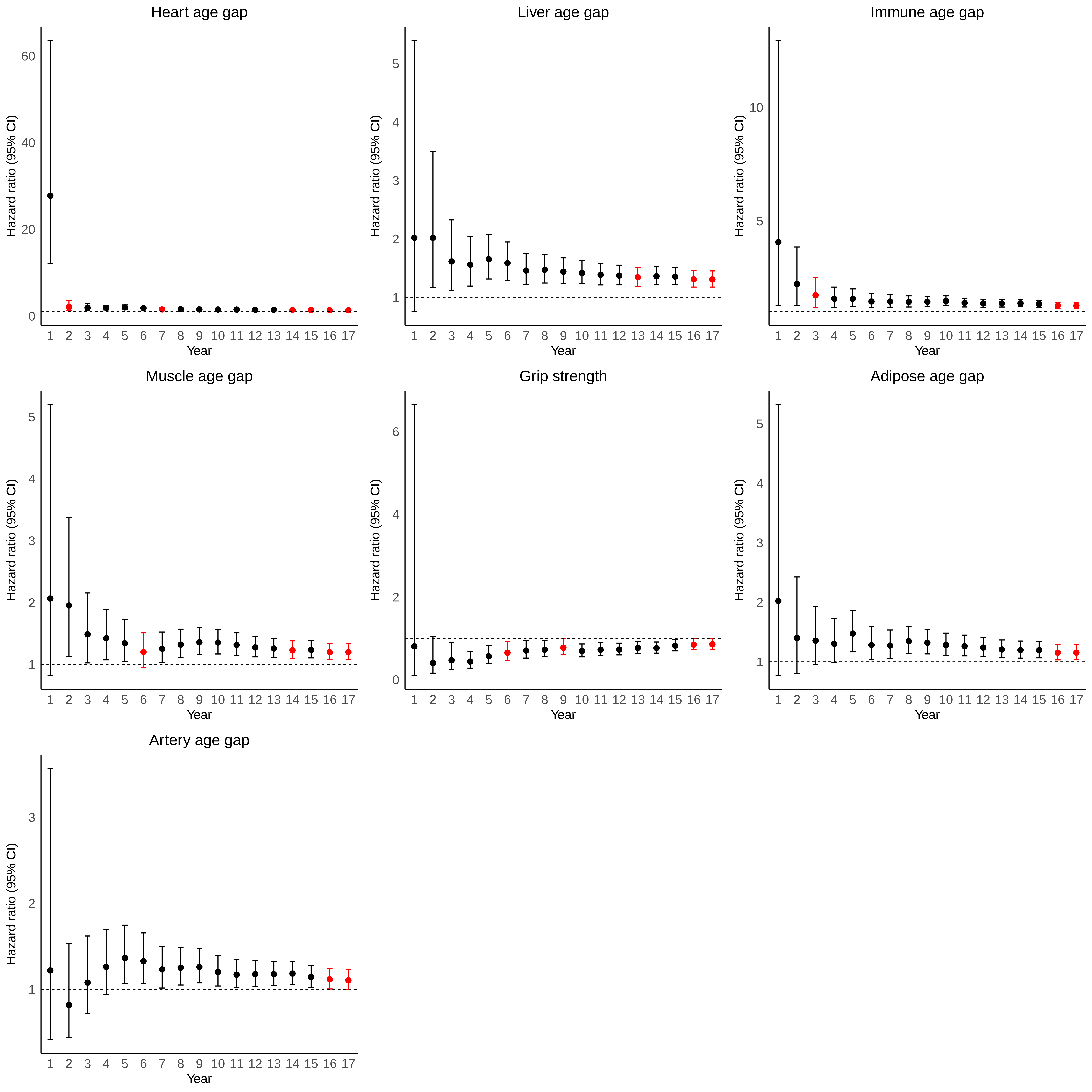
Heart age gap
Liver age gap
Immune age gap
Muscle age gap
Grip strength
Adipose age gap
Artery age gap
Hazard ratio (95% CI)
Year
