# Supplementary material for: Multimodal Ageing Biomarkers and Plasma Proteomic Signatures Associated With All‐Cause Mortality
Source: Aging Cell. 2026 Oct 2;25(10):e70747. doi: 10.1111/acel.70747 (PMC13631562; doi:10.1111/acel.70747)
Supplement: Supplementary file 1 [file ACEL-25-e70747-s001.zip › ACEL_70747__pe.docx]

**Manuscript title: Multimodal Ageing Biomarkers and Plasma Proteomic Signatures Associated with All‐Cause Mortality**

**SUPPLEMENTARY TABLE LEGENDS**

**SUPPLEMENTARY TABLE 1.** Quality control status of SomaScan 11K plasma proteins in the LBC1936. The 11,083 measured aptamers were classified as (1) passing quality control, (2) being non-human targets, (3) likely to be affected by pre-analytical variation, (4) having a high coefficient of variation, (5) having a high relative fluorescence unit signal, or (6) having a signal-to-noise ratio <1.

**SUPPLEMENTARY TABLE 2.** Associations of individual ageing biomarkers with all-cause mortality in the LBC1936. Cox proportional hazards regression results are presented for associations between individual biomarkers and 16-year all-cause mortality, with incremental adjustment for demographic variables, modifiable lifestyle risk factors, and seven medical conditions.

**SUPPLEMENTARY TABLE 3.** Associations of all ageing biomarkers with all-cause mortality in the LBC1936. Cox proportional hazards regression results are presented for a model including all ageing biomarkers simultaneously and adjusted for demographic variables and modifiable lifestyle risk factors.

**SUPPLEMENTARY TABLE 4.** Associations of individual plasma proteins with all-cause mortality in the LBC1936. Cox proportional hazards regression results are presented for associations between individual plasma proteins and 16-year all-cause mortality, with incremental adjustment for demographic variables, modifiable lifestyle risk factors, and seven medical conditions.

**SUPPLEMENTARY TABLE 5.** Elastic net-derived weights for the 37 plasma protein predictors of all-cause mortality in the LBC1936.

**SUPPLEMENTARY FIGURE LEGENDS**

**SUPPLEMENTARY FIGURE 1**

**Characterisation of plasma proteomic organ ageing signatures in the LBC1936. (A)** Predicted plasma proteomic ages for eleven organs in LBC1936 wave 2 participants (N = 795). Each point represents an individual, and the black dashed line indicates the mean organ age. **(B)** Density plots of organ age gaps, defined as standardised residuals (mean = 0, standard deviation = 1) from the linear regression of predicted organ age on chronological age. Positive values, indicating accelerated ageing, are shown in pink, and negative values, indicating slower ageing, are shown in green. **(C)** Pairwise Pearson correlation matrix of organ age gaps. Only significant correlations (P_FDR_ < 0.05) are shown. Positive correlations are shown in pink, and negative correlations in green. **(D)** Number and percentage of individuals with extreme ageing in one or more organs. Extreme ageing is defined as an organ age gap exceeding ±2 standard deviations from the population mean in at least one organ.

**SUPPLEMENTARY FIGURE 2**

**Comparison of all-cause mortality associations for 23 ageing biomarkers using maximum versus common sample sizes.** Associations between 23 ageing biomarkers measured at wave 2 and 16-year all-cause mortality were estimated using age- and sex-adjusted Cox proportional hazards models in the LBC1936. Log hazard ratios derived from the maximum available sample for each biomarker (N = 656-861) are plotted against those obtained from the common subset with complete data across all biomarkers (N = 460).

**SUPPLEMENTARY FIGURE 3**

**Correlations among ageing biomarkers and modifiable lifestyle risk factors in the LBC1936.** Pairwise Pearson correlations among 23 ageing biomarkers and modifiable lifestyle risk factors measured at wave 2 (N = 460). Red indicates positive correlations and green indicates negative correlations, with colour intensity reflecting correlation strength. Only significant associations are shown (P_FDR_ < 0.05).

**SUPPLEMENTARY FIGURE 4**

**Explanatory power for all-cause mortality across different ageing biomarker domains.** Nagelkerke’s pseudo-R² was calculated to assess the relative explanatory power of Cox proportional hazards models incorporating different combinations of predictors in the LBC1936. All biomarkers were measured at wave 2 (N = 460), and all-cause mortality was assessed over an average of 16 years of follow-up. Eight models were fitted with the following predictor sets: (1) demographics; (2) demographics + lifestyle; (3) demographics + lifestyle + GrimAge2; (4) demographics + lifestyle + organ clocks; (5) demographics + lifestyle + physical function; (6) demographics + lifestyle + brain measures; (7) demographics + lifestyle + four significant biomarkers; and (8) demographics + lifestyle + 21 ageing biomarkers. Demographic factors included age and sex, and lifestyle factors included smoking, alcohol consumption, and body mass index (BMI). Organ clocks comprised the 11 plasma proteomic clocks. Physical function measures included grip strength, respiratory function, and walking time. Brain measures included MRI-derived total brain volume, white matter hyperintensity volume, brain age gap, and general cognitive function. The four significant biomarkers were total brain volume, white matter hyperintensity volume, general cognitive function, and walking time, as these remained independently associated with mortality after adjustment for all other ageing biomarkers.

**SUPPLEMENTARY FIGURE 5**

**Change in ageing biomarker associations with all-cause mortality over 17 years of follow-up in the LBC1936.** Cox proportional hazards models were used to examine associations between individual ageing biomarkers measured at wave 2 and all-cause mortality. Models were fitted annually across 17 years of follow-up and adjusted for chronological age, sex, lifestyle risk factors, and seven medical conditions. Forest plots show the seven ageing biomarkers that violated the proportional hazards assumption. Years with significant violations (Schoenfeld P < 0.05) are highlighted in red. Circles represent hazard ratios and error bars indicate 95% confidence intervals. The horizontal dotted line denotes a hazard ratio of 1.

**SUPPLEMENTARY FIGURE 6**

**Change in plasma protein associations with all-cause mortality over 17 years of follow-up in the LBC1936.** Cox proportional hazards models were used to examine associations between individual SomaScan 11K plasma proteins measured at wave 2 and all-cause mortality. Models were fitted annually across 17 years of follow-up and adjusted for chronological age, sex, lifestyle risk factors, and seven medical conditions. Forest plots show the 10 proteins among the top 20 mortality-associated proteins that violated the proportional hazards assumption. Years with significant violations (Schoenfeld P < 0.05) are highlighted in red. Circles represent hazard ratios and error bars indicate 95% confidence intervals. The horizontal dotted line denotes a hazard ratio of 1.
