# Supplementary material for: Multimodal Ageing Biomarkers and Plasma Proteomic Signatures Associated With All‐Cause Mortality
Source: Aging Cell. 2026 Oct 2;25(10):e70747. doi: 10.1111/acel.70747 (PMC13631562; doi:10.1111/acel.70747)
Supplement: Supplementary file 1 [file ACEL-25-e70747-s001.zip › FigureS1.pdf]

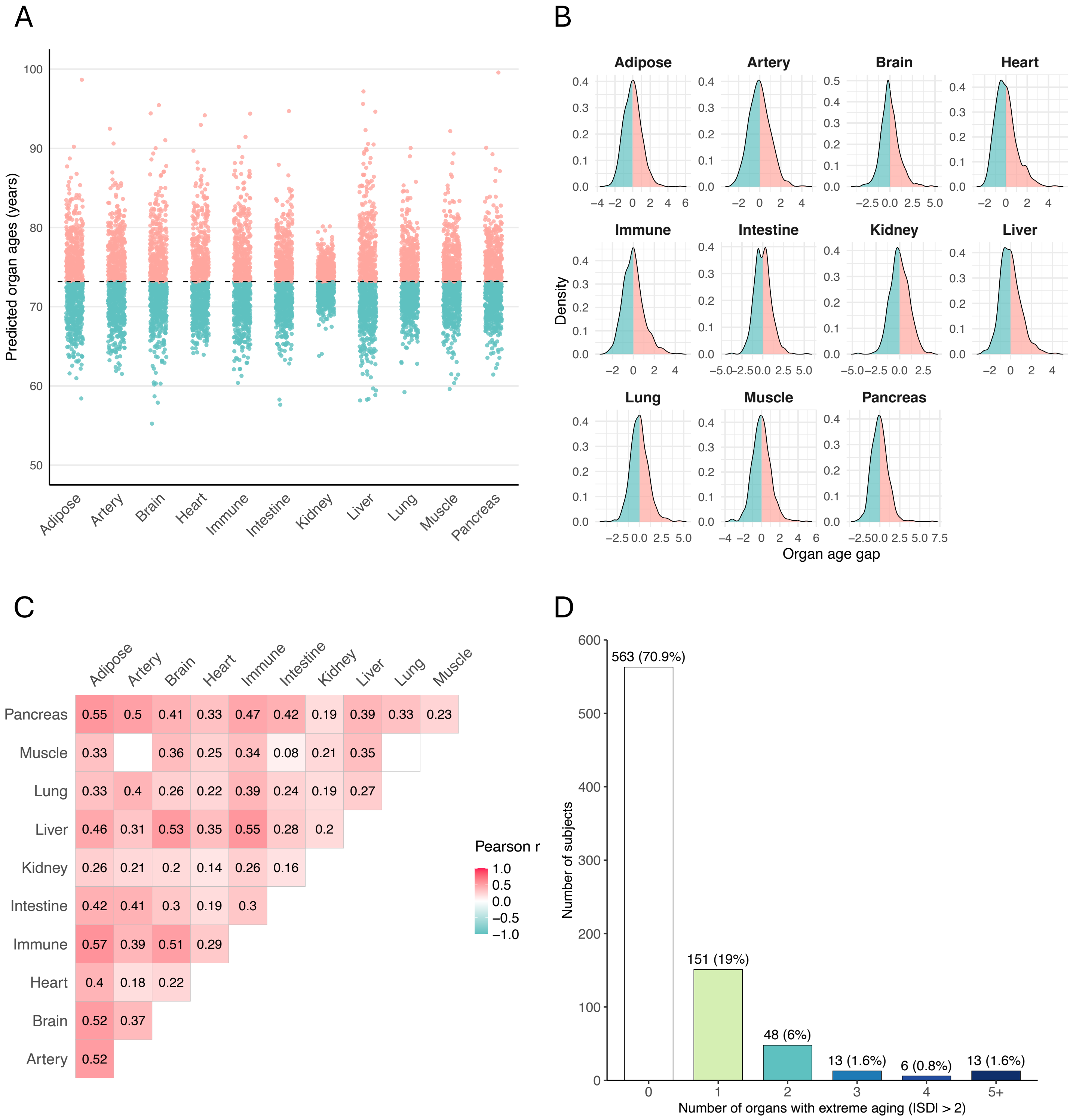
A
Predicted organ ages (years)
100
90
80
70
60
50
Adipose
Artery
Brain
Heart
Immune
Intestine
Kidney
Liver
Lung
Muscle
Pancreas
B
Adipose
Artery
Brain
Heart
Immune
Intestine
Kidney
Liver
Lung
Muscle
Pancreas
Density
Organ age gap
C
Adipose
Artery
Brain
Heart
Immune
Intestine
Kidney
Liver
Lung
Muscle
Pancreas 0.55 0.5 0.41 0.33 0.47 0.42 0.19 0.39 0.33 0.23
Muscle 0.33 0.36 0.25 0.34 0.08 0.21 0.35
Lung 0.33 0.4 0.26 0.22 0.39 0.24 0.19 0.27
Liver 0.46 0.31 0.53 0.35 0.55 0.28 0.2
Kidney 0.26 0.21 0.2 0.14 0.26 0.16
Intestine 0.42 0.41 0.3 0.19 0.3
Immune 0.57 0.39 0.51 0.29
Heart 0.4 0.18 0.22
Brain 0.52 0.37
Artery 0.52
Pearson r
1.0
0.5
0.0
−0.5
−1.0
D
Number of subjects
600
500
400
300
200
100
0
563 (70.9%)
151 (19%)
48 (6%)
13 (1.6%)
6 (0.8%)
13 (1.6%)
0
1
2
3
4
5+
Number of organs with extreme aging (ISDI > 2)
