# Supplementary material for: Multimodal Ageing Biomarkers and Plasma Proteomic Signatures Associated With All‐Cause Mortality
Source: Aging Cell. 2026 Oct 2;25(10):e70747. doi: 10.1111/acel.70747 (PMC13631562; doi:10.1111/acel.70747)
Supplement: Supplementary file 1 [file ACEL-25-e70747-s001.zip › FigureS2.pdf]

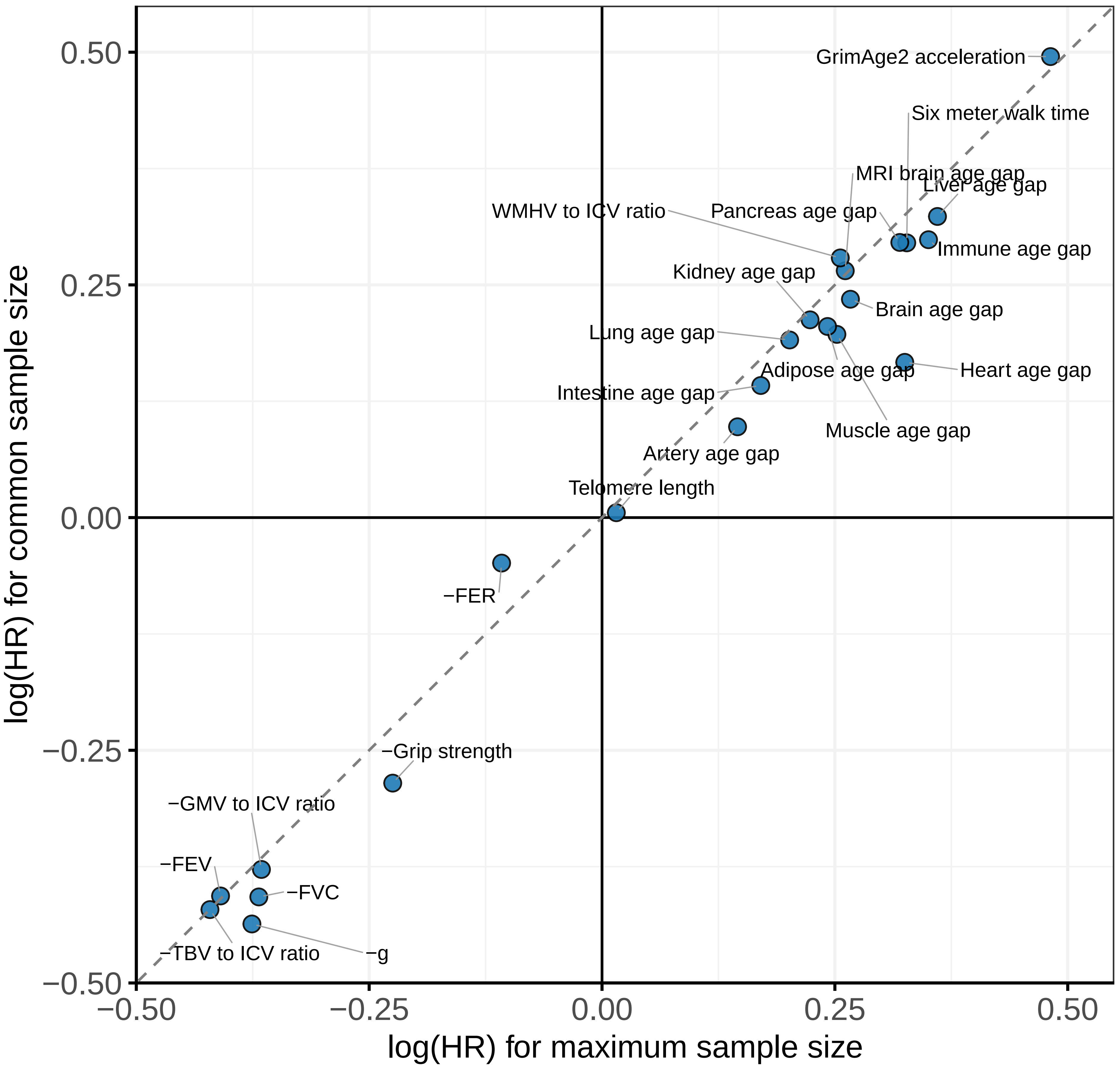

GrimAge2 acceleration
Six meter walk time
MRI brain age gap
Liver age gap
WMHV to ICV ratio
Pancreas age gap
Immune age gap
Kidney age gap
Brain age gap
Lung age gap
Heart age gap
Adipose age gap
Intestine age gap
Muscle age gap
Artery age gap
Telomere length
−FER
−Grip strength
−GMV to ICV ratio
−FEV
−FVC
−TBV to ICV ratio
−g
0.50
0.25
0.00
−0.25
−0.50
log(HR) for common sample size
log(HR) for maximum sample size
