# Supplementary material for: Multimodal Ageing Biomarkers and Plasma Proteomic Signatures Associated With All‐Cause Mortality
Source: Aging Cell. 2026 Oct 2;25(10):e70747. doi: 10.1111/acel.70747 (PMC13631562; doi:10.1111/acel.70747)
Supplement: Supplementary file 1 [file ACEL-25-e70747-s001.zip › FigureS4.pdf]

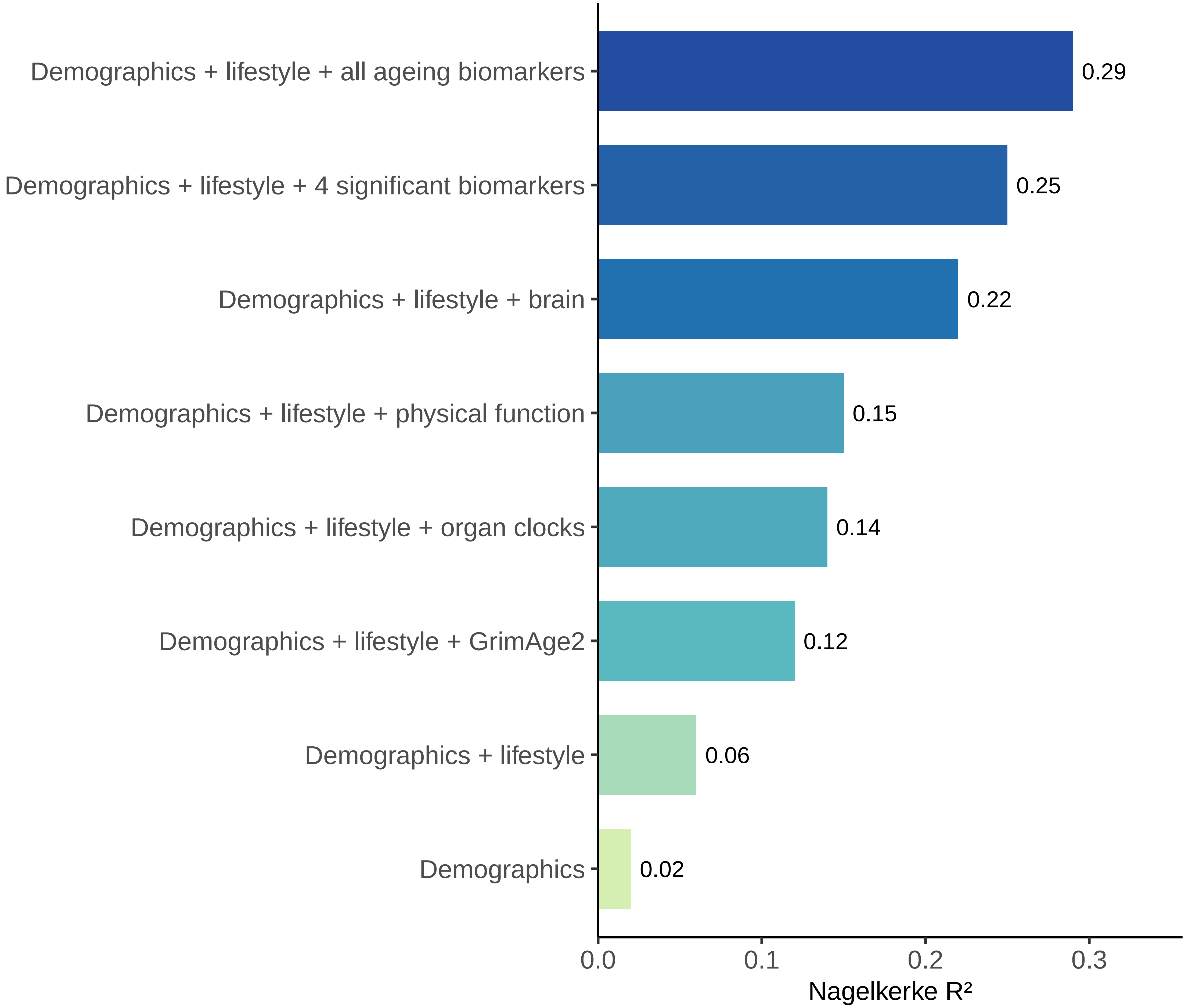
Demographics + lifestyle + all ageing biomarkers
0.29
Demographics + lifestyle + 4 significant biomarkers
0.25
Demographics + lifestyle + brain
0.22
Demographics + lifestyle + physical function
0.15
Demographics + lifestyle + organ clocks
0.14
Demographics + lifestyle + GrimAge2
0.12
Demographics + lifestyle
0.06
Demographics
0.02
0.0
0.1
0.2
0.3
Nagelkerke R²
